# PeERF1, a SHINE-Like Transcription Factor, Is Involved in Nanoridge Development on Lip Epidermis of *Phalaenopsis* Flowers

**DOI:** 10.3389/fpls.2019.01709

**Published:** 2020-01-30

**Authors:** Pei-Han Lai, Li-Min Huang, Zhao-Jun Pan, Wann-Neng Jane, Mei-Chu Chung, Wen-Huei Chen, Hong-Hwa Chen

**Affiliations:** ^1^ Department of Life Sciences, National Cheng Kung University, Tainan, Taiwan; ^2^ Institute of Ecology and Evolutionary Biology, National Taiwan University, Taipei, Taiwan; ^3^ Institute of Plant and Microbial Biology, Academia Sinica, Taipei, Taiwan; ^4^ Orchid Research and Development Center, National Cheng Kung University, Tainan, Taiwan; ^5^ Institute of Tropical Plant Sciences, National Cheng Kung University, Tainan, Taiwan

**Keywords:** AP2/EREBP, cutin biosynthesis genes, lip, nanoridge, orchid, *Phalaenopsis*, transcription factor

## Abstract

*Phalaenopsis* orchids have a spectacular floral morphology with a highly evolved lip that offers a landing platform for pollinators. The typical morphological orchid lip features are essential for the special pollination mechanism of *Phalaenopsis* flowers. Previously, we found that in the lip, a member of the AP2/EREBP protein family was highly expressed. Here, we further confirmed its high expression and characterized its function during lip development. Phylogenetic analysis showed that AP2/EREBP belongs to the Va2 subgroup of ERF transcription factors. We named it PeERF1. We found that *PeERF1* was only expressed at stage 5, as flowers opened. This coincided with both thickening of the cuticle and development of nanoridges. We performed knockdown expression of *PeERF1* using CymMV-based virus-induced gene silencing in either the *AP2* conserved domain, producing *PeERF1_AP2*-silenced plants, or the *SHN* specific domain, producing *PeERF1_SHN-*silenced plants. Using cryo-SEM, we found that the number of nanoridges was reduced only in the *PeERF1_AP2*-silenced group. This change was found on both the abaxial and adaxial surfaces of the central lip lobe. Expression of *PeERF1* was reduced significantly in *PeERF1_AP2*-silenced plants. In cutin biosynthesis genes, expression of both *PeCYP86A2* and *PeDCR* was significantly decreased in both groups. The expression of *PeCYP77A4* was reduced significantly only in the *PeERF1_AP2*-silenced plants. Although *PeGPAT* expression was reduced in both silenced plants, but to a lesser degree. The expression of *PeERF1* was significantly reduced in the petal-like lip of a big-lip variant. *PeCYP77A4* and *PeGPAT* in the lip were also reduced, but *PeDCR was not*. Furthermore, heterologous overexpression of *PeERF1* in the genus *Arabidopsis* produced leaves that were shiny on the adaxial surface. Taken together, our results show that in *Phalaenopsis* orchids PeERF1 plays an important role in formation of nanoridges during lip epidermis development.

## Introduction

*Phalaenopsis* orchids are renowned for their unique and elegant floral morphology and long florescence duration. Recently, they have become the model Orchidaceae research plants. Two databases for genetic information have been established, OrchidBase 3.0 and Orchidstra 2.0 (Fu et al., 2011; Su et al., 2013a; Tsai et al., 2013; Chao et al., 2017). The floral morphology of *Phalaenopsis* orchids includes three sepals, three petals, and one column. The column is formed by fusion of the style and a part of the androecium. The outer two perianth whorls are typically petaloid and are referred to as tepals. Instead of producing three uniform petals, *Phalaenopsis* flowers have a highly evolved, modified, and resupinated inner medium petal, the lip (Rudall and Bateman, 2002; Tsai et al., 2004; Tsai and Chen, 2006; Tsai et al., 2008; Pan et al., 2011; Pan et al., 2014). This lip is understood to play an important role both in pollination and evolution (Robinson and Burns-Balogh, 1982; Cozzolino and Widmer, 2005; Mondragón-Palomino and Theißen, 2008), as it provides a platform for pollinators.

**Figure 1:**
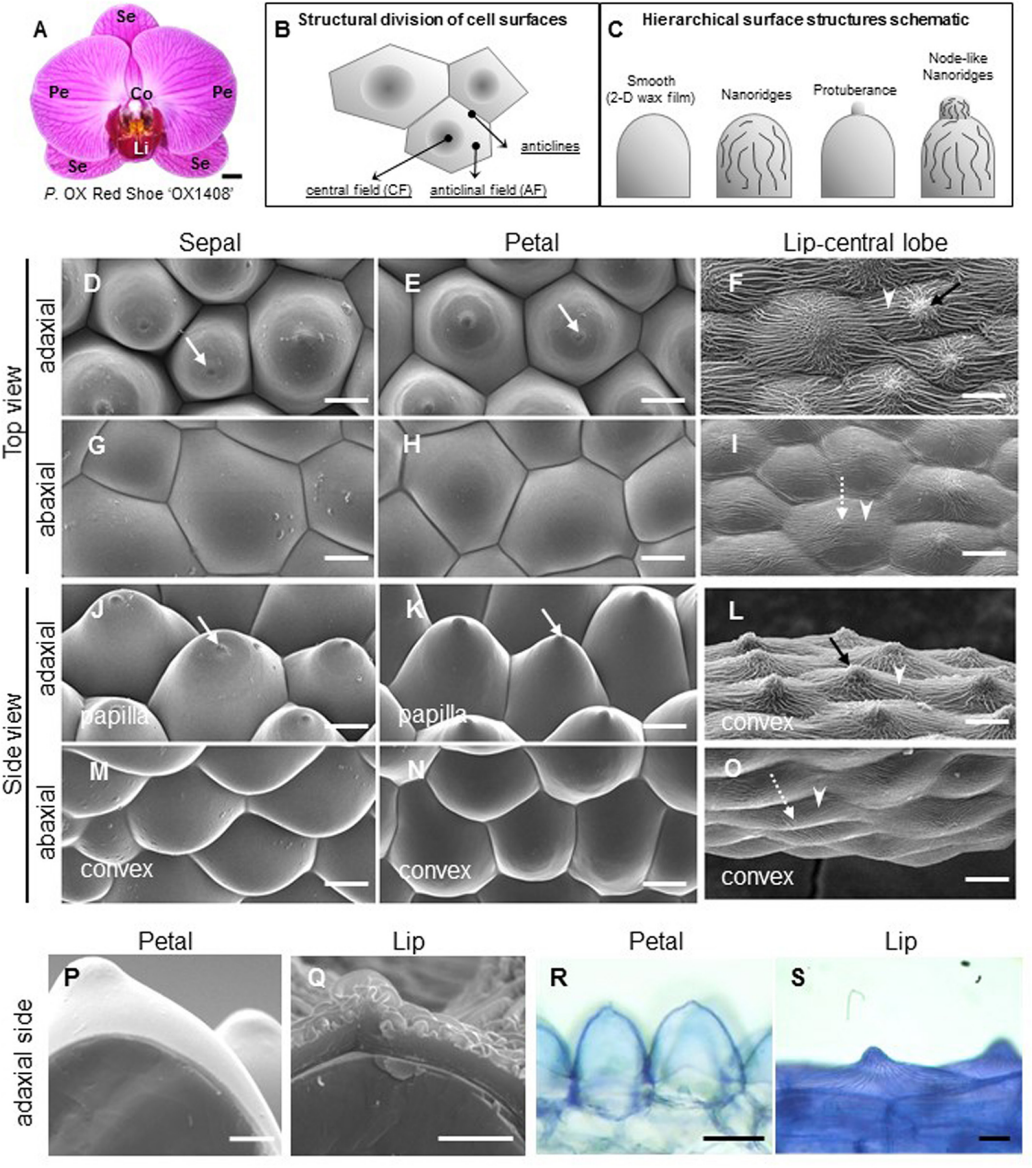
Ultrastructure of floral epidermal cells in the cultivar *P.* OX Red Shoes “OX1408.” **(A)** Floral morphology. Scale bar, 5 cm. Se, sepal; Pe, petal; Li, lip; Co, column. **(A)** Schematic diagrams of the cell structural division of a single cell surface. The central field (CF) is in the center middle of the cell surface and the anticline field (AF) is between the middle area and the boundary of the cell. Anticlines directly connected to the cell area (modified after Koch et al., 2009a). **(A)** Schematic diagrams of epidermis that built up the cell hierarchical structures with 2-D smooth wax films or decorated with protuberance, nanoridges, and node-like nanoridges. **(D**–**O)** Cyro-SEM top view and side view of epidermal cells of perianth organs. White arrows indicate a protuberance on the top of the cell. Black arrows indicate node-like nanoridges in the CF of the cell. White arrowheads indicate parallel nanoridges in the AF of the cell. Scale bars, 30 μm. **(P**–**Q)** Cross section of adaxial epidermal cells of petal and lip. Scale bars, 10 μm. **(R**–**S)** Lipophilic dye “Sudan Black B” staining on the adaxial epidermal cells of petal and lip. Scale bars, 100 μm.

Lip morphogenesis consists of five stages, from the embedded stage 1 to the open flower of stage 5. There is no division of the lip at stage 1. However, in stage 2, the lip divides quickly into three distinct parts. There are two lateral lobes, one central lobe, and one callus. The split lip forms a tunnel-like structure in the mature flower (**Figure 1A**, Li), a feature related to evolved pollination strategies (Cozzolino and Widmer, 2005). Although *Phalaenopsis* orchids exhibit unique floral morphological features, Cryo-scanning electron microscopy (Cryo-SEM) has revealed that the perianth lip epidermis has a unique functional morphology not found in sepals or petals (Pan et al., 2011; Hsieh et al., 2013a; Hsieh et al., 2013b; Pan et al., 2014; Hsu et al., 2015b). Heavy and dense nanoridges cover the lip epidermis. These undulated nanostructures, also called cuticular folds, are assumed to contain cuticular lipids (Koch et al., 2008; Koch et al., 2009a; Koch et al., 2009b).

Diverse perianth and floral epidermis adaptations have evolved in both eudicots and monocots. In many flowering plants, the epidermal surfaces of sepals and petals display a range of patterns in combination with diverse micro- and nanostructures (Kay et al., 1981; Whitney et al., 2009; Whitney et al., 2011b; Kourounioti et al., 2013). More than 75% of petal epidermal cells of angiosperms are conical or papillate, usually on the adaxial side where potential pollinators would be found (Kay et al., 1981; Whitney et al., 2009; Whitney et al., 2011a; Whitney et al., 2011b). Moreover, in many plants, sepal and petal epidermal cells are covered with various density and orientation of nanoridges (Jeffree, 2006). These structures on the surface of sepal and petal epidermal cells are believed to attract pollinators and enhance pollination success through visual signals (Whitney et al., 2009; Whitney et al., 2011b; Kourounioti et al., 2013; Moyroud et al., 2017) and act as tactile signals affecting pollinator movement (Prüm et al., 2011; Rands et al., 2011; Prüm et al., 2012; Prüm et al., 2013; Adachi et al., 2015). Moreover, cell surface cuticle structures can strengthen cells and thereby function many ways in plant development as well as survival and defense in unfavorable environments, such as under biotic or abiotic stress (e.g., dehydration, pathogens, UV light, frost, and insect attacks) (Koch et al., 2008; Koch et al., 2009a; Koch et al., 2009b).

The first identified transcription factors (TFs) that regulate cuticle biosynthesis are SHINEs/WAX INDUCERs (SHNs/WINs), members of the V group of the ethylene responsive factor (ERF) subfamily of the apetala2/ethylene response element binding protein (AP2/EREBP) TF family (Aharoni et al., 2004; Broun et al., 2004; Nakano et al., 2006). In *Arabidopsis SHINE* gain-of-function mutant (*shn*) and plants overexpressing *AtWIN1/SHN1*, *AtSHN2*, or *AtSHN3* have shiny leaves and increased accumulation of epidermal wax on the top of leaves as compared with wild type (Aharoni et al., 2004; Broun et al., 2004). By co-silencing all three AtSHN clade members, SHNs redundantly regulate the formation of petal surface nanoridges and also cell elongation, adhesion, and separation (Shi et al., 2011). Recently, increased number of SHN-like TFs that belong to the ERF-V group have been identified and they exhibit various functions during plant physiological processes.

The ERF-V group includes two subgroups: Va and Vb. The Va subgroup contains two conserved motifs of the conserved middle motif (CMV-1) and C-terminal motif (CMV-2), which the Vb subgroup does not contain (Nakano et al., 2006). The ERF-Va subgroup is further divided into two subgroups, Va1 and Va2, containing a complete or incomplete CMV-1 motif, respectively (Nakano et al., 2014). Functional characterization of genes in the Va1 subgroup from several plants indicates that they are involved in cuticle development. *Arabidopsis* AtSHNs regulate cuticle formation (Aharoni et al., 2004; Broun et al., 2004; Shi et al., 2011).

Similar research has been done with several plant species. In barley it was found that HvNud is involved in the lipid biosynthesis of the grain surface, which produces hulled caryopses (Taketa et al., 2008). Tomato SlSHINE3 is involved in cutin metabolism of fleshy fruit epidermal cells for patterning the epidermal surface (Shi et al., 2013) and SlSHN1 is involved in wax accumulation of leaf epidermal cells, which enhances drought tolerance (Al-Abdallat et al., 2014). Rice OsAP2/ERF-”N-22” is involved in wax biosynthesis and also enhances drought resistance (Mawlong et al., 2014), while wheat TdSHN1 is involved in the cuticle formation of leaf surfaces (Jäger et al., 2015). *Eucalyptus* EgrSHN1 and EgrSHN2 are involved in cell wall biosynthesis of flowers (Marques et al., 2013). Hence, the complete CMV-1 and CMV-2 motifs of ERF-Va1 genes are deemed the SHINE domains. SHINE domains are considered to be important in cuticle development. The subgroup Va1 is identified as the SHINE clade (Aharoni et al., 2004).

In contrast, the Va2 subgroup, with an incomplete CMV-1 motif, is involved in various physiological processes: *Arabidopsis* At5g25190 is induced by 1-aminocyclopropane-1-carboxylicacid (ACC) and salt (NaCl) and was named the ethylene- and salt-inducible ERF gene (*AtESE3*) (Zhang et al., 2011), but its overexpression confers no typical SHINE phenotype (Aharoni et al., 2004). Tomato LeERF1 regulates fruit ripening and softening (Li et al., 2007). *Populus* PtaERF003 is involved in lateral root formation (Trupiano et al., 2013). *Eucalyptus* Egr33m and Egr40m are involved in wood cell wall biosynthesis (Marques et al., 2013). Tomato SlERF52 regulates flower pedicel abscission (Nakano et al., 2014). Whereas, NvERF045 in berries regulates berry ripening and is also involved in cuticle development (Leida et al., 2016). Moreover, the Vb subgroup At5g19790 does not contain CMV-1 and CMV-2 motifs and is important in low potassium signaling (Kim et al., 2012).

Gene associated with cutin biosynthesis for epidermal nanoridge formation include CYP86A and CYP77A, members of a cytochrome P450 family, glycerol-3-phosphate acyltransferase 6 (GPAT), and defective in cuticular ridges (DCR) (Kannangara et al., 2007; Li-Beisson et al., 2009; Panikashvili et al., 2009; Shi et al., 2011; Shi et al., 2013; Petit et al., 2016; Mazurek et al., 2017). GPAT6 and CYP77A6 are for the formation of floral cutin in *Arabidopsis thaliana* (Li-Beisson et al., 2009). *CYP86A4* has been reported as one of the downstream target genes *SHN* (Shi et al., 2011; Shi et al., 2013). It has been shown that *DCR*-deficient plants have defective cuticle formation with altered epidermal cell differentiation (Panikashvili et al., 2009). This defective formation in reproductive and vegetative tissues was correlated with low abundance of 9(10),16-dihydroxyhexadecanoic acid in the cutin polymer of *DCR* (*At5g23940*)-deficient plants (Panikashvili et al., 2009).

We previously identified several unigenes dominantly expressed in the *Phalaenopsis* lip (Hsiao et al., 2013). Among them, one member of the AP2 family, *P. equestris ethylene responsive factor 1* (*PeERF1*), was found to be most similar to the SHINE clade homolog At5g25190.

Here, to extend our understanding of the function of *PeERF1* in *Phalaenopsis* orchids, we analyzed its spatial and temporal gene expression; downregulated *PeERF1* expression by using CymMV-based virus-induced gene silencing (VIGS) in orchids; and examined heterologous overexpression of *PeERF1* in *Arabidopsis* for comparison. The abnormal phenotypes of nanoridge sculpture patterns of lip epidermal cells were observed in the somaclonal variant, *P.* “Join Big foot TH365” containing enlarged petal-like lip mutants. We further investigated the relationship of putative *Phalaenopsis* orthologs of known cutin biosynthetic genes with the cuticle formation in *Phalaenopsis* lips. These genes include two cytochrome P450s, *PeCYP86A2* and *PeCYP77A4*, and two putative acyltransferases, *PeGPAT* and *PeDCR*. We hope that these results will contribute to the understanding of transcriptional regulation of late-stage orchid lip formation during floral morphogenesis.

## Materials and Methods

### Plant Materials

We conducted gene spatial expression analysis on specimens of *Phalaenopsis equestris* obtained from the Taiwan Sugar Corp. (Tainan, Taiwan), and both gene temporal expression and VIGS experiments on the commercial cultivar *P.* OX Red Shoes “OX1408” obtained from Oxen Biotechnology Corp. (Tainan, Taiwan).

The flower buds of *P.* OX Red Shoes “OX1408” were divided into five stages by size (**Figure 3C**). A single raceme spike inflorescence embraces 8–10 flowers. The smallest flower bud is embedded in the tip of an inflorescence and is stage 1 (< 0.5 cm), followed by successive stage 2 (0.5–1 cm), stage 3 (1–2 cm), stage 4 (2–3 cm), and blooming flowers are stage 5 (floral diameter of 12 cm) (**Figure 3C**). For comparison, we used wild-type flower and big-lip variant flower (petal-like lip) of *P*. hybrid “Join Big foot” (*P*. Yu Pin Easter Island x *P*. I- Hsin Diamond “Join White of Love”). These were provided by Join Orchids Incorporation (Tainan, Taiwan). For VIGS experiments, plants were kept in the greenhouse at the Tainan District Agricultural Research and Extension Station, Council of Agriculture, under a controlled temperature of 27°C/22°C (day/night). Other mature orchid plants were maintained in the greenhouse at National Cheng Kung University under natural light and controlled temperature from 23°C to 27°C.

### Cryo-SEM

We examined changes in cellular morphology from the 1^st^ to the 8^th^ blooming flowers of silenced plants (stage 5, floral diameter of 12 cm) using Cryo-SEM. Sample preparation and Cryo-SEM examination follows previous research (Hsieh et al., 2013a; Pan et al., 2014). Fresh samples were dissected and loaded on the stub, which was subsequently frozen with liquid nitrogen slush, and then quickly transferred to a sample preparation chamber at -160°C for 5 min. After that time the temperature was raised to -85°C and sublimed for 15 min. Samples were then coated with platinum (Pt) at -130°C and transferred to the cryo-stage in an SEM chamber and observed at -160°C using Cryo scanning electron microscope (FEI Quanta 200 SEM/Quorum Cryo System PP2000TR FEI) with 20 kV.

Images were taken under a Cryo stage at < -160°C. We measured the nanoridge area on 40 flowers, 5 flowers from each of 8 plants using ImageJ (http://rsb.info.nih.gov/ij/). Mean data were compared by Duncan’s multiple-range test, using SPSS v17.

### Cloning and Characterization of *PeERF1*

Using TRIsure reagent (Bioline, UK) total RNA was extracted and then treated with RNase-free DNaseI (Invitrogen, USA) to remove residual DNA. We cloned the full-length cDNA of *PeERF1* (accession no. MG948436) using a SMART rapid amplification of cDNA ends (RACE) kit (Clontech, USA). We randomly selected 6 to 8 positive clones for sequencing. For gene expression analysis, quantitative real-time RT-PCR (qRT-PCR) was performed in triplicate and repeated independently three times as previously described (Hsu et al., 2015a). Primers for all the PCR and qRT-PCR experiments are in **Supplementary Table S1**.

For qRT-PCR, the cDNA template was mixed with 2X SYBR Green PCR master mix (Applied Biosystems, Norwalk, CT, USA) in an ABI 7300 instrument (Applied Biosystems) with three biological replicates. For gene quantification, qRT-PCR was performed at stage 5 flowers of *PeERF1*-silenced plants in triplicate, and repeated in three silenced plants independently. For PCR reaction, each sample was analyzed in triplicate. Reactions involved incubation at 50°C for 2 min, then 95°C for 10 min, and thermal cycling for 40 cycles (95°C for 15 s and 60°C for 1 min). The relative quantification was calculated according to the manufacturer’s instructions (Applied Biosystems). To control the integrity of RNA and normalize target RNA copy numbers in gene-silenced and mock-treated flowers, the housekeeping gene *PeActin4* (AY134752) was recruited as an internal control for normalization (Chen et al., 2005).

## Sequence Alignment and Phylogenetic Analysis

Multiple sequence alignment was generated by using AlignX (Vector NTI advance 11, Invitrogen). The protein sequences of SHN homologous TFs were obtained from the National Center for Biotechnology Information (NCBI) databases (http://www.ncbi.nlm.nih.gov/), and accession numbers are as follows: *A. thaliana* AtSHN1 (NP_172988), AtSHN2 (NP_196680), AtSHN3 (NP_851073), At5g25190 (NP_197901.1), and At5g19790 (NP_197480.1); tomato (*Solanum lycopersicum*) SlSHN1 (XP_004235965), SlSHN3 (XP_004240977), and SlERF52 (BAO18577); tomato (*Lycopersicon esculentum*) LeERF1 (AAL75809); berry (*Vitis vinifera*) VvERF045 (ANT73695); barley (*Hordeum vulgare L.*) HvNud (BAG12386); wheat (*Triticum aestivum L.*) TdSHN1 (ANY98960); rice (*Oryza sativa*) OsAP2/ERF-”N-22” (ACU44657), and *Populus* (*P. tremula*x *P. alba*) PtaERF003 (Potri.018G021900). *Eucalyptus grandis* SHN homologous TFs can be accessed in the Phytozome database (http://www.phytozome.net/cgi-bin/gbrowse/eucalyptus/): EgrSHN1 (Eucgr.C04221.1), EgrSHN2 (Eucgr.C01178.1), Egr33m (Eucgr.C02719.1), and Egr40m (Eucgr.C03947.1) (Marques et al., 2013). These sequences were used to construct phylogenetic trees by using MEGA5.0 (Tamura et al., 2011). Phylogenetic relationships were inferred by the neighbor-joining method and evolutionary distances were computed by the Poisson correction method. Bootstrap values were calculated with 1,000 replicates.

### Virus-Induced Gene Silencing (VIGS)

VIGS experiments with *PeERF1* were performed as previously described (Hsieh et al., 2013a; Pan et al., 2014); 142-nt and 175-nt fragments for the conserved AP2 domain and the specific incomplete “SHINE domains,” i.e., CMV-1 and CMV-2 motifs of *PeERF1*, respectively, were constructed into the pCymMV-Gateway plasmid (Lu et al., 2007). The constructed VIGS-silencing plasmids for producing the *PeERF1_AP2*-silenced and *PeERF1_SHN-*silenced plants were named pCymMVGateway-PeERF1_AP2 and pCymMV-Gateway-PeERF1_SHN, respectively. These plasmids were transformed into *Agrobacterium* (strain EHA105). For infiltration, *Agrobacterium tumefaciens* strain EHA105 containing pCymMVGateway-PeERF1_AP2 or mMV-Gateway-PeERF1_SHN were grown overnight at 28°C to OD_600_ = 1. After centrifugation, bacterial cell pellets were resuspended by adding 300 μl MS medium containing 100 μM acetosyringone and allowed to stand at room temperature for 0.5 h. Two methods of Agro-infiltration were used: inflorescence injection and leaf injection. For inflorescence injection, suspensions were injected into the stalk of the raceme with eight internodes and one visible floral bud (extruding out of its bract) by use of a 1-ml syringe with a needle in all silencing treatments. The raceme stalk usually emerges from the stem between the third and fourth leaves. For leaf injection, suspensions were injected into the leaf directly above the emerging inflorescence. Mock-treated plants were recruited as the negative control. They were handled the same and contained an empty vector of a *Cymbidium* mosaic virus infectious clone with a Gateway system vector. Transformed EHA105 was injected into both inflorescence spikes and the leaf directly above the emerging inflorescence. For VIGS, eight independent *PeERF1*_AP2-silenced and eight *PeERF1*_ SHN-silenced plants as well as eight mock control plants were generated, and repeated twice independently. qRT-PCR was used to examine the knockdown expression of cuticle biosynthesis-related genes in the 5^th^ floral buds (stage 3, length of 1–2 cm) after agro-infiltration in triplicate and repeated three times independently; Cryo-SEM was used to examine the changes in cellular morphology from the 5^th^ to the 8^th^ blooming flowers of silenced plants (stage 5, floral diameter of 12 cm).

### Ectopic Expression of *PeERF1* in *Arabidopsis*

*A. thaliana* ecotype Columbia was used for transformation experiments as previously described (Chen et al., 2012). Full-length cDNA of *PeERF1* was cloned into the pBI121 vector under the control of the constitutive *Cauliflower* mosaic virus (CaMV) 35S promoter, and the resulted plasmid was named pBI121-*PeERF1*. We then introduced pBI121-*PeERF1* plasmid into *Agrobacterium tumefaciens* (strain GV3101) and transformed into wild-type *Arabidopsis* by the floral dip method (Clough and Bent, 1998). In total, 60 kanamycin-resistant T1 seedlings were obtained and grown at 23°C in a growth chamber under long-day conditions (16-h light/8-h dark). A total of 457 T2 heterozygous seedlings were obtained with a segregation ratio of 3:1 as analyzed by chi-square test. For gene expression analysis, RNA samples of T1 heterozygous plants were extracted to confirm the successful expression by qRT-PCR in triplicate and repeated three times independently. The primers were listed in **Supplementary Table S1**.

## Results

### *Phalaenopsis* Flowers Showed Unique Lip Cuticle Features Relative to Other Perianth Epidermal Morphology

To investigate the detailed ultrastructure of orchid floral epidermal cells, we first examined the floral epidermal morphology of a native species, *P. aphrodite* subsp. *formosana* (**Supplementary Figure S1** ) and the commercial cultivar, *P.* OX Red Shoe “OX1408” (**Figure 1A**) by using Cryo-SEM. The schematic diagrams of different regions and hierarchical structures on the cell surface of *Phalaenopsis* flowers are shown in **Figures 1B**, **C**. The “central field” (CF) and “anticlinal field” (AF) represent the inner and outer parts of cells, respectively, and the boundaries of two perpendicular cell walls are “anticlines” (**Figure 1B**). The epidermal cells of the *Phalaenopsis* orchid perianth have a variety of cell morphology, including convex or papilla cell shapes. The cell surface of the perianth epidermal cells can be smooth, covered with 2-D wax films or decorated with protuberances, nanoridges, or other node-like nanoridges (**Figure 1C**).

The adaxial epidermal cells of sepals and petals in *P*. OX Red Shoe “OX1408” flowers featured a papilla cell shape with a protuberance on the top (**Figures 1D**, **E**, **J**, **K**, white arrow), and the abaxial epidermal cells of sepals and petals had a convex cell shape with smooth 2-D wax films (**Figures 1G**, **H**, **M**, **N**). In contrast, heavy and dense nanoridges were found on the lip epidermis. The lip adaxial epidermal cells had convex cell shape with the appearance of node-like nanoridges in the CF (**Figures 1F**, **L**, black arrow) and parallel and radial nanoridges in the AF (**Figures 1F**, **L**, white arrowhead) of cell surfaces. The lip abaxial epidermal cells had a convex cell shape with parallel nanoridges in the CF (**Figures 1I**, **O**, white dashed arrow) and AF of cell surfaces (**Figures 1I**, **O**, white arrowhead). The same heavy nanoridges were also observed on the lip epidermis of *P. aphrodite* subsp. *formosana* (**Supplementary Figure S1D**).

Cross sections of petal and lip adaxial epidermal cells showed markedly different thickness of cuticles (**Figures 1P**, **Q**). The petal adaxial epidermal cells were covered with smooth 2-D wax films (**Figure 1P**) as compared with the complex and heavy nanoridges on the lip adaxial epidermal cells (**Figure 1Q**). Histochemistry staining with the lipophilic dye “Sudan Black B” revealed a lipid layer on the petal cuticle and lip adaxial epidermal cells (**Figures 1R**, **S**).

### *PeERF1* Displayed Lip Development-Associated Gene Expression Patterns

**Figure 2:**
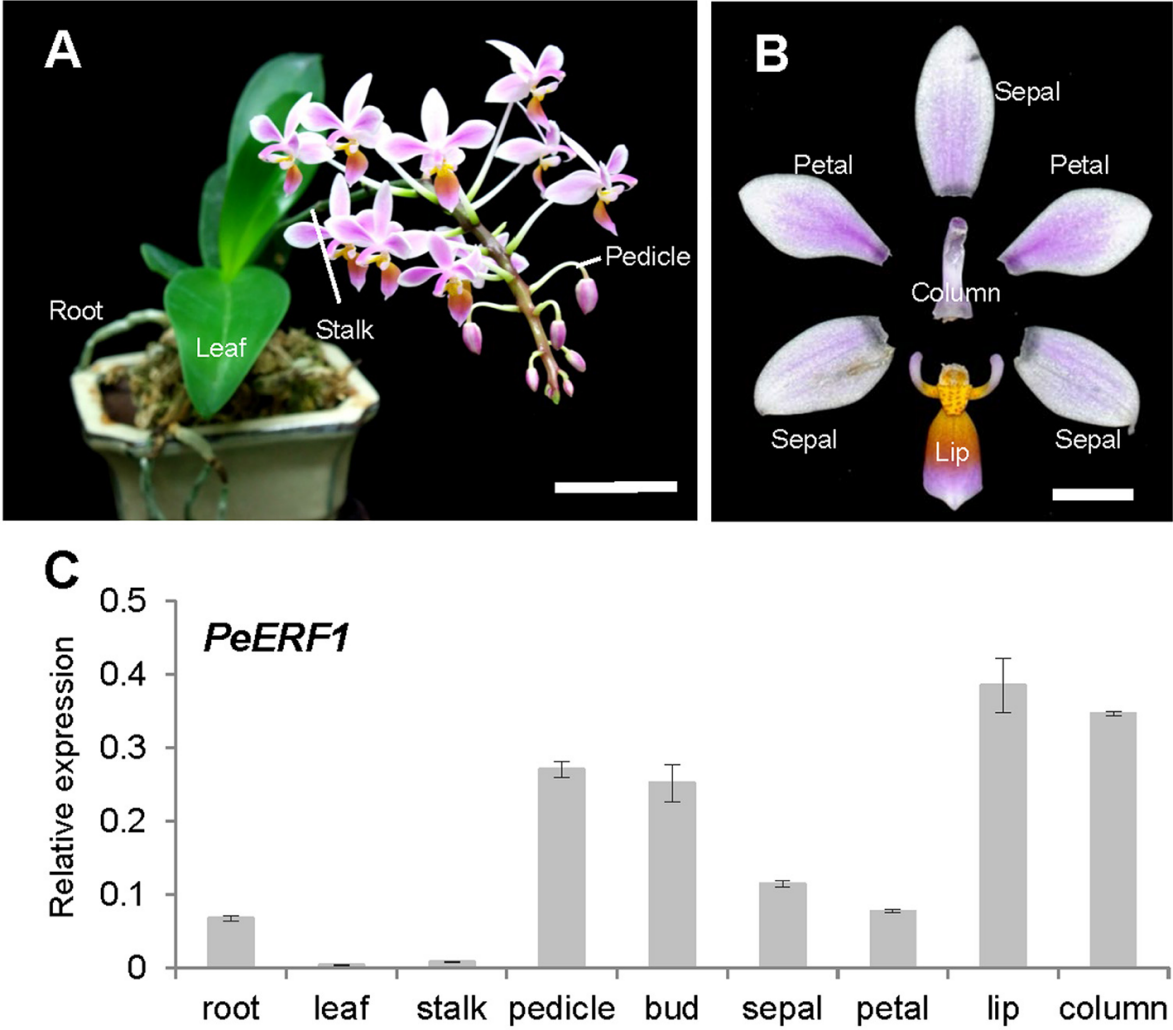
Spatial expression patterns of *PeERF1* in the native species *P. equestris*. **(A)** Various vegetative and reproductive tissues were analyzed, including roots, leaves, pedicles, and stalks. Scale bar, 5 cm. **(B)** Flower organs were analyzed, including sepals, petals, lip, and column. Scale bar, 1 cm. **(C)** Spatial expression patterns of *PeERF1* in various organs. Total RNA were extracted from variant tissues in three independent plants. Three technical repeats were performed for each sample. Data are mean ± SD. Numbers above the bars are expression levels after normalization with the internal control (*PeActin4*) (Chen et al., 2005).

We analyzed and confirmed the spatial expression patterns of *PeERF1* in both vegetative (root, leaf, and stalk) and reproductive organs (pedicle, bud, sepal, petal, lip, and column) of *P. equestris* (**Figures 2A, B**) by qRT-PCR. *PeERF1* was highly expressed in reproductive organs (pedicle and flower bud) with lower expression in roots, and very low expression in leaf and stalk tissue. As expected, *PeERF1* was highly expressed in the lip and column, with threefold expression in lip as compared with sepals and petals (**Figure 2C**).

### Development of Cuticles on Lip Epidermal Cells Concomitant With the *PeERF1* Gene Expression at Late Stage of Lip Morphogenesis

**Figure 3:**
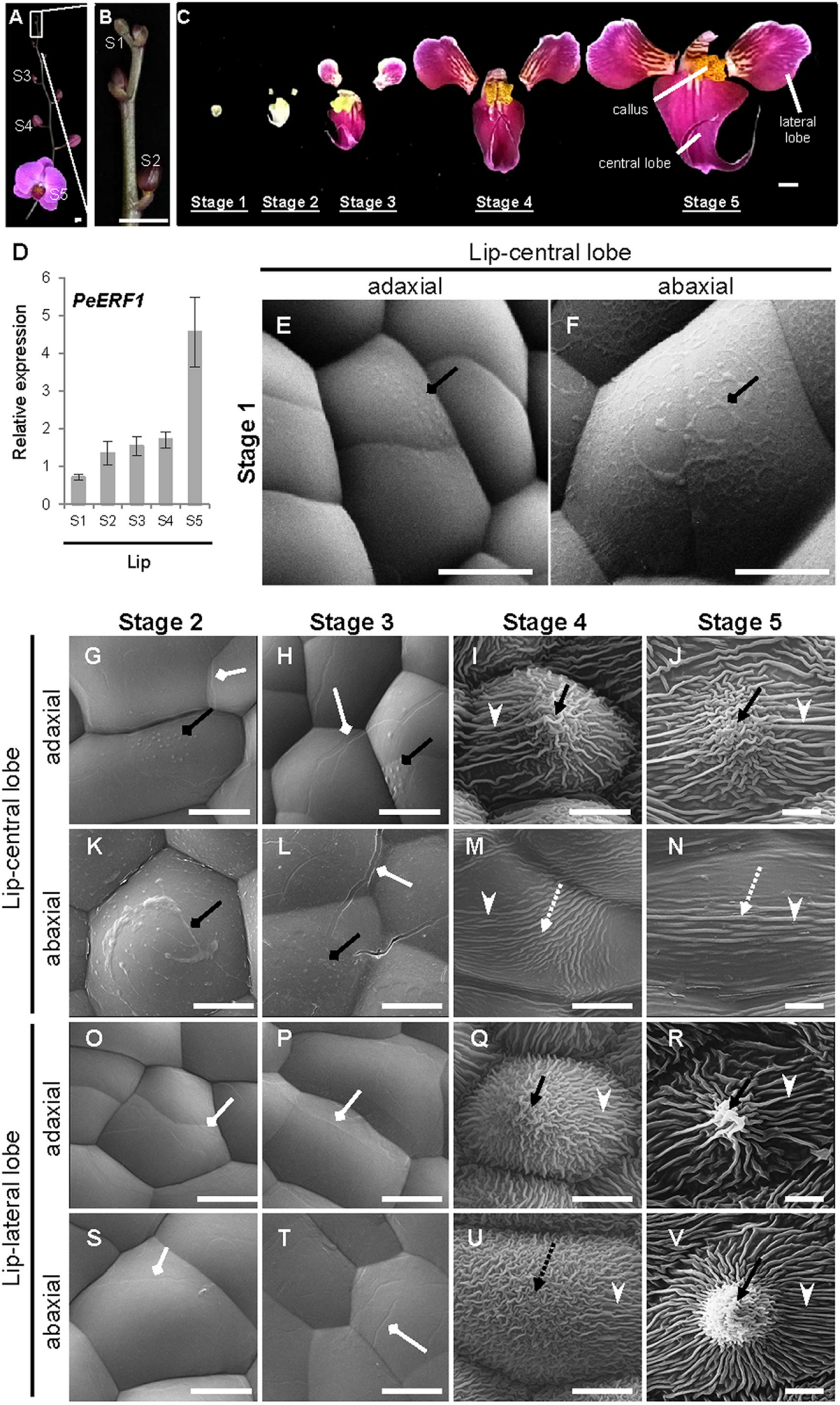
Temporal expression patterns of *PeERF1* and the ultrastructure of lip epidermal cells during various developmental stages of *P.* OX Red Shoes “OX1408” flowers. **(A**–**C)** Various stages of lip development during floral morphogenesis. S1, stage 1, flower bud (0–0.5 cm); S2, stage 2, flower bud (0.5–1 cm); S3, stage 3, flower bud (1–2 cm); S4, stage 4, flower bud (2–3 cm); S5, stage 5, flowering. Scale bars, 2 cm **(A**–**B)** and 1 cm **(C)**. **(D)** Temporal expression patterns of *PeERF1* at various lip developmental stages. Total RNA were extracted from the various lip developmental stages in three independent plants. Three technical repeats were performed for each sample. Data are mean ± SD. Numbers above the bars are expression levels after normalization with the internal control (*PeActin4*). **(E**–**V)** Cyro-SEM of adaxial and abaxial sites of epidermal cells of different parts of lip organs during developmental stages. Black rhombus-head lines indicate the secreted bubble-like preliminary cuticle components. White rhombus-head lines indicate the traces of preliminary nanoridges. Black arrows indicate node-like nanoridges in the CF of the cell. Black dashed arrows indicate irregular nanoridges in the CF of the cell. White arrowheads indicate parallel nanoridges in the AF of the cell. Scale bars, 10 μm.

Temporal expression of *PeERF1* during lip morphogenesis was examined in five stages of lip development (**Figures 3A–C**). *PeERF1* showed low and increasing expression from stage 1 to stage 4, with a sharp increase at stage 5 (**Figure 3D**).

The ultrastructure of the lip epidermis during various developmental stages was examined under Cryo-SEM. Both adaxial and abaxial surfaces of epidermal cells in the lip-central lobe showed secreted bubble-like preliminary cuticle components and heavy cuticle layers from stages 1 to 3 (**Figures 3E**, **F**, **G**, **H**, **K**, **L**, black rhombus-head line). Traces of preliminary nanoridges started to form on the lip epidermis from stages 2 to 3 (**Figures 3G**, **H**, **L**, **O**, **P**, **S**, **T**, white rhombus-head line). These then thickened and the number of nanoridges increased from stages 4 to 5 (**Figures 3I**, **J**, **M**, **N**, **Q**, **R**, **U**, **V**).

The final mature forms of nanoridges varied in different parts of the lip. The adaxial epidermal cells of the central lobe showed anode-like nanoridges in the CF (**Figure 3J**, black arrow), and parallel and radial nanoridges in the AF (**Figure 3J**, white arrowhead). Similar nanoridges on adaxial epidermal cells of the central lobe were also found on the adaxial and abaxial epidermal cells of lateral lobes (**Figure 3R**, **V**). In contrast, the abaxial epidermal cells of the central lobe showed looser parallel nanoridges in the CF (**Figure 3N**, white dashed arrow) and AF (**Figure 3N**, white arrowhead). Thus, the typical lip epidermal features containing nanoridges are important markers of the lip morphological identity. These results suggest that *PeERF1* may have a correlation with nanoridge formation during lip morphogenesis.

### PeERF1 is Phylogenetically Assigned to the Va2 Subgroup of the ERF Subfamily

**Figure 4:**
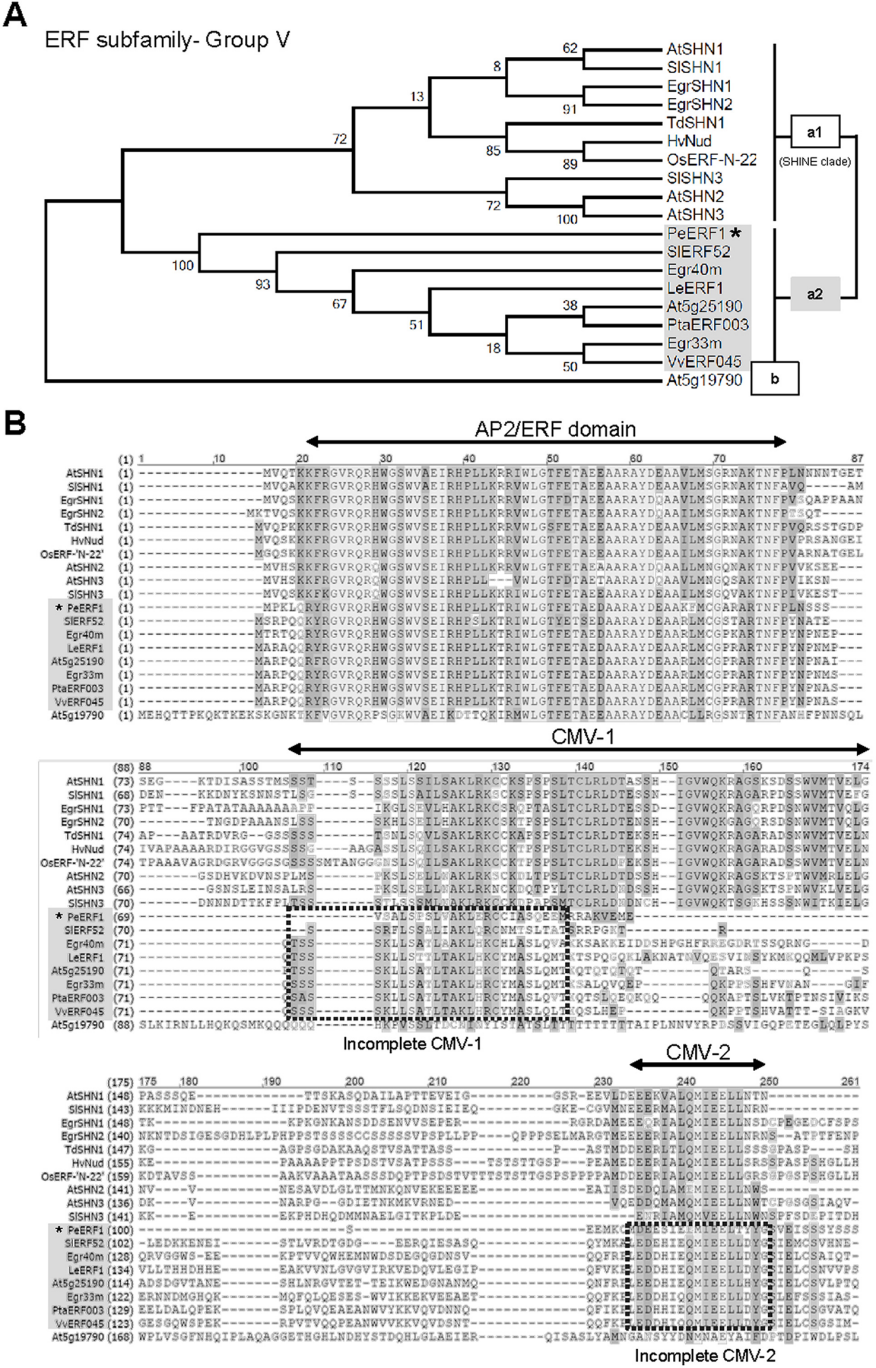
Phylogenetic analysis and sequence alignment of PeERF1. **(A)** Phylogenetic analysis of PeERF1 with published ERF-V group proteins. Bootstrap values were calculated with 1,000 replicates. PeERF1 is highlighted with a star (*). **(B)** Multiple alignment of amino acid sequences of PeERF1 and ERF-V group proteins. According to the existence of “SHINE domains” (CMV-1 and CMV-2 motifs), the ERF-V group was classified into two groups: Va group, with SHINE domains, and Vb group, without SHINE domains. In addition, the Va group was further divided into two subgroups: Va1 subgroup, with complete SHINE domains (deemed as “SHINE clade”), and Va2 subgroup, with incomplete SHINE domains.

Phylogenetic analysis showed that PeERF1 belongs to the Va2 subgroup of the ERF subfamily of the AP2/EREBP family (**Figure 4A**), and the sequence alignment indicated that PeERF1 contains incomplete “SHINE domains” (CMV-1 and CMV-2 motifs) (**Figure 4B**). Of note, the protein sequences of incomplete “SHINE domains” of PeERF1 are distinguished from other members of the ERF-Va2 subgroup, which results in a separate branch from the other ERF-Va2 members and closer to the ERF-Va1 subgroup (**Figure 4**).

### Silencing of *PeERF1* Resulted in Abnormal Sculpture of Nanoridges on the Lip Epidermis

To assess the role of *PeERF1* in lip cuticle development, CymMV-based VIGS was used to silence its expression with a 142-nt conserved AP2 domain (139–281 nt) and the 175-nt incomplete “SHINE domains” (346–521 nt) in *P.* OX Red Shoes “OX1408.” This resulted in plants that were *PeERF1*_*AP2*-silenced and *PeERF1*_*SHN-*silenced (**Supplementary Figure S2A**). Using Cryo-SEM we examined the perianth epidermis micro-morphology from *PeERF1*_*AP2*-silenced plants. Lip epidermis nanoridges showed continuously altered distribution of structures within one plant from the 1^st^ to the 7^th^ flowers. We also found more severely altered phenotypes, with looser, thinner, uneven, and hollowed distribution of nanoridge structures especially on the lip epidermis of the 6^th^ and the 7^th^ flowers (**Supplementary Figure S3**). Flowers on these silenced plants showed no obvious difference in floral morphology as compared with mock-treated plants (**Supplementary Figures S2B–D**).

**Figure 5:**
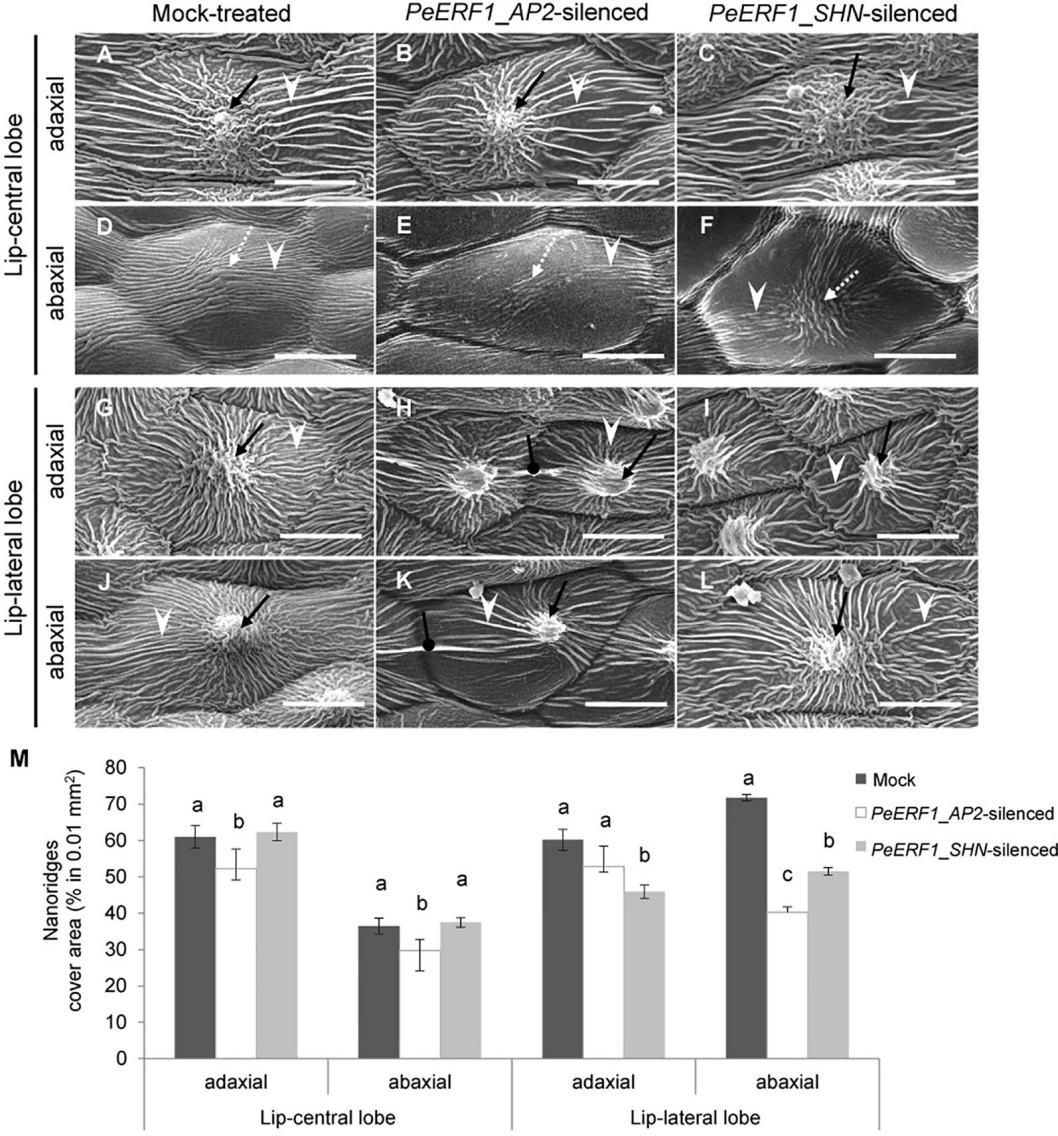
Nanoridge characteristics of ultrastructure of lip epidermal cells from 7^th^ blooming flowers of *PeERF1-*silenced *P.* OX Red Shoes “OX1408.” **(A**–**L**) Cryo-SEM was used to examine the changes in cellular morphology from the 5^th^ to the 8^th^ blooming flowers of silenced plants (stage 5, floral diameter of 12 cm). Top view of adaxial and abaxial sites of lip central and lateral lobe epidermis in mock-treated, *PeERF1_*AP2*-*silenced, and *PeERF1_*SHN*-*silenced plants. Black arrows indicate node-like nanoridges in the CF of the cell. White dashed arrows indicate parallel nanoridges in the CF of the cell. White arrowheads indicate parallel nanoridges in the AF of the cell. Black spherical-head line indicates thicker and extended nanoridges across anticlines of cells. Scale bars, 30 μm. **(M)** The coverage of nanoridges on the top of adaxial and abaxial lip epidermal cells of mock-treated and *PeERF1*-silenced flowers. For Cryo-SEM examination, eight plants were injected with control, specific domain and conserved domain separately, and repeated twice. Data are mean ± SD (n = 15); the same letters above the bars indicate no statistical difference by Duncan's multiple range test (P < 0.05).

The most severe phenotype was observed on the 7^th^ blooming flowers in *PeERF1*_*AP2*-silenced plants (**Figure S3D**). This was concomitant with the expression of *PeERF1* at late stage of lip morphogenesis. We then further examined the cell surface on the lip epidermis of the 7^th^ blooming flowers of both types of silenced plants (**Figures 5A–L**, top view; **Supplementary Figure S4**, side view). Looser and fewer nanoridges in the AF of adaxial and abaxial surfaces of central lobe epidermal cells were observed as compared with mock-treated plants (**Figures 5A–F**, white arrowhead). In addition, the AF showed uneven distribution of parallel and irregular nanoridges, which was hollow by reducing the coverage of nanoridges on adaxial and abaxial surfaces of lateral lobe epidermal cells (**Figures 5H, I, K, L**, white arrowhead; **Figure 5M**). Moreover, *PeERF1*_*AP2*-silenced plants showed unique, thicker, and extended nanoridges across anticlines of both the adaxial and abaxial surface of lip-lateral lobe epidermal cells (**Figures 5H**, **K**, black spherical-head line).

In contrast, the sepals and petals of all blooming flowers of *PeERF1*-silenced plants otherwise showed no obvious phenotypic changes in the adaxial and abaxial surface of epidermal cells (**Supplementary Figure S5**). *PeERF1* and cutin biosynthesis genes were downregulated in both silenced plants; however, these cells do not have cuticle on their cell surface (**Supplementary Figure S6**).

### Off-Target Silencing Effects in VIGS Phenotype

**Figure 6:**
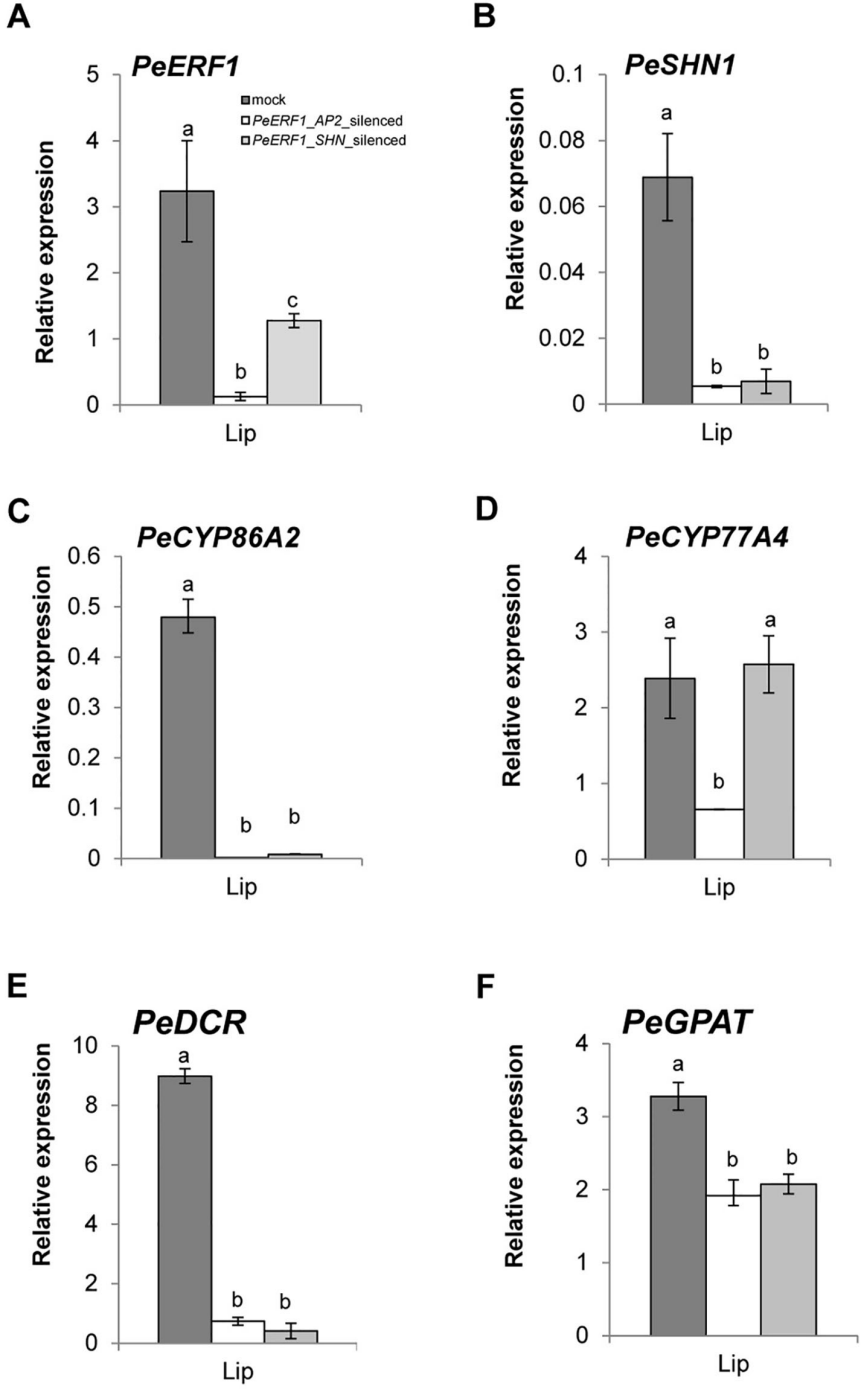
Expression patterns of *PeERF1* and cuticle-associated genes in *PeERF1*-silenced plants. Transcript level of *PeERF1* **(A)**, *PeSHN1* **(B)**, and cutin metabolism-related genes (*PeCYP86A2*, *PeCYP77A4*, *PeGPAT*, and *PeDCR*) **(C**–**F)** in mock-treated and *PeERF1*-silenced flowers. Total RNA of sepal, petal, and lip were extracted from the 5^th^ floral bud (stage 3, length of 1–2 cm) at 30 days post-inoculation. Three technical repeats and three biological repeats were performed for gene expression analysis in the *PeERF1_*silenced plants. Data are mean ± SD; the same letters above the bars indicate no statistical difference by Duncan's multiple range test (P < 0.05).

Both *PeERF1*-silenced plants showed downregulation of *PeERF1* (**Figures 6A** and **S6A**). However, the transcript level of *PeSHN1* was much lower than that of *PeERF1* (**Figures 6A**, **B**). We speculate that the downregulation of *PeSHN1* was due to off-target effect since there are 71.8% and 47.1% identity between the AP2 and SHN domains of *PeERF1* and *PeSHN1* genes, respectively (**Supplementary Figure S7**). However, it is also possible that transcription of *PeSHN1* is regulated by PeERF1 to some extent (**Figures 6A, B** and **Supplementary Figure S6B**).

### Reduced Expression of Cutin Biosynthesis Genes in the *PeERF1-*Silenced Plants

Next, we examined whether the expression of cuticle biosynthesis genes was altered upon the reduction of *PeERF1* expression in the VIGS plants. Expression of *PeERF1* in the lip was reduced significantly in *PeERF1_AP2*-silenced plants, but less so in *PeERF1_SHN*-silenced plants. *PeCYP86A2* and *PeCYP77A4*, *PeGPAT*, *PeGPAT*, and *PeDCR* are putative *Phalaenopsis* orthologs of known cutin biosynthetic genes, which contribute to the cuticle formation in *Phalaenopsis* lips. Similar to the reduced expression of *PeERF1* in the *PeERF1_SHN*-silenced plants, the expressions of *PeCYP86A2, PeDCR*, and *PeCYP77A4* were reduced significantly in *PeERF1_AP2*-silenced plants (**Figures 6C, D**). The expressions of *PeCYP86A2* and *PeDCR* were reduced, while the expression of *PeCYP77A4* was not affected in the *PeERF1_SHN*-silenced plants (**Figures 6C, D**). The expression of *PeGPAT* was reduced in both *PeERF1_AP2*-silenced and *PeERF1_SHN-*silenced plants, but to a less extent (**Figure 6F**). Therefore, both *PeCYP77A4* and *PeGPAT* were found to be crucial for cuticle formation, and their expressions were regulated by other TFs in addition to PeERF1 or PeSHN1.

### Nanoridges Disappeared in the Petal-Like Lip of the “Join Big Foot” Variant

**Figure 7:**
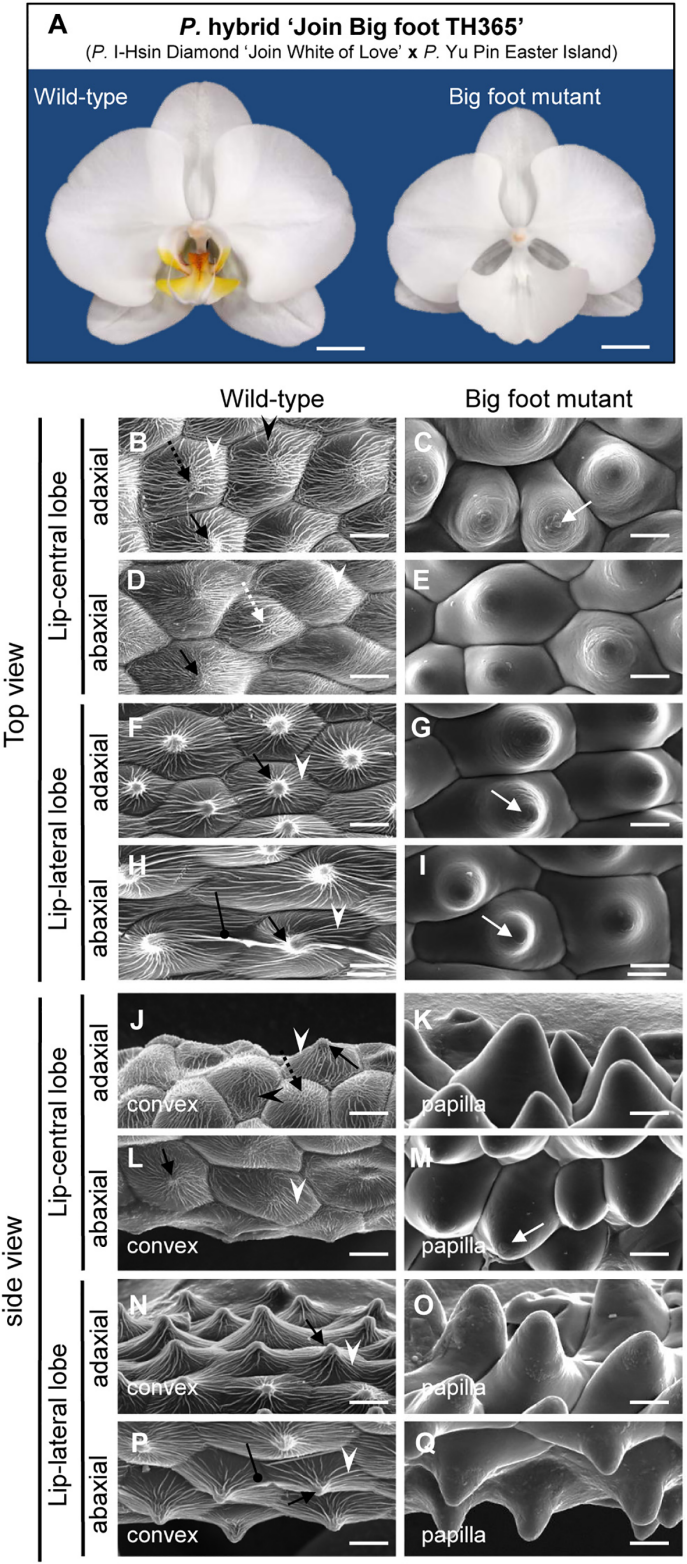
Floral morphology and ultrastructure of lip epidermal cells of somaclonal variants with normal lip or enlarged petal-like lip mutants. **(A)** The flowers of somaclonal variants of *P.* hybrid “Join Big foot TH365” (*P.* I-Hsin Diamond “Join White of Love” x *P.* Yu Pin Easter Island). The lip morphology of “big foot mutant” flower showed morphological conversions to petal-like structure. Scale bars, 2 cm. **(B**–**Q)** Top view and side view of adaxial and abaxial sites of lip central and lateral lobe epidermis in “wild-type” and “big foot mutant” of somaclonal variants of *P.* “Join Big foot TH365” flowers. White arrows indicate a protuberance on the top of the cell. Black arrows indicate node-like nanoridges in the CF of the cell. White dashed arrows indicate parallel nanoridges in the CF of the cell. Black dashed arrows indicate irregular nanoridges in the CF of the cell. White arrowheads indicate parallel nanoridges in the AF of the cell. Black arrowheads indicate irregular nanoridges in the AF of the cell. Black spherical-head line indicates thicker and extended nanoridges across anticlines of the cells. Different cell shape types of floral epidermal cells are in the lower left corner of each panel. Scale bars, 30 μm.

From population of the *P.* hybrid “Join Big foot” grown from seedlings we selected two plants each with wild-type flower (normal split-lip organ) and with the big-lip enlarged petal-like lip flower. We observed these in order to confirm the association of *PeERF1* and cuticle formation during lip morphogenesis (**Figure 7A**). Cryo-SEM revealed that cell morphology and ultrastructure were severely changed in the petal-like lip epidermal cells of the big-lip variant as compared with wild type (**Figures 7B–Q**). In wild type, the epidermal cells of split-lip organs showed an abnormal, uneven, and hollowed distribution of parallel nanoridges in the AF of cells (**Figures 7B, D, F, H**). In the big-lip variant, all adaxial and abaxial surfaces of the lip epidermal cells displayed similar characteristics of petal adaxial epidermal cells with a papilla cell shape with smooth 2-D wax films and a protuberance on the top of the cell (**Figures 7C, E, G, I, K, M, O, Q**; **Supplementary Figures S8** and **S9**). The phenotypic observations were done in three big-foot variant plants. Lip morphology was altered to various levels, from severe to mild, yet the phenotypic change for nanoridge formation was the same (data not shown).

### Reduced Expression of *PeERF1* and Cutin Biosynthesis Genes Associated With Big-Foot Variant

**Figure 8:**
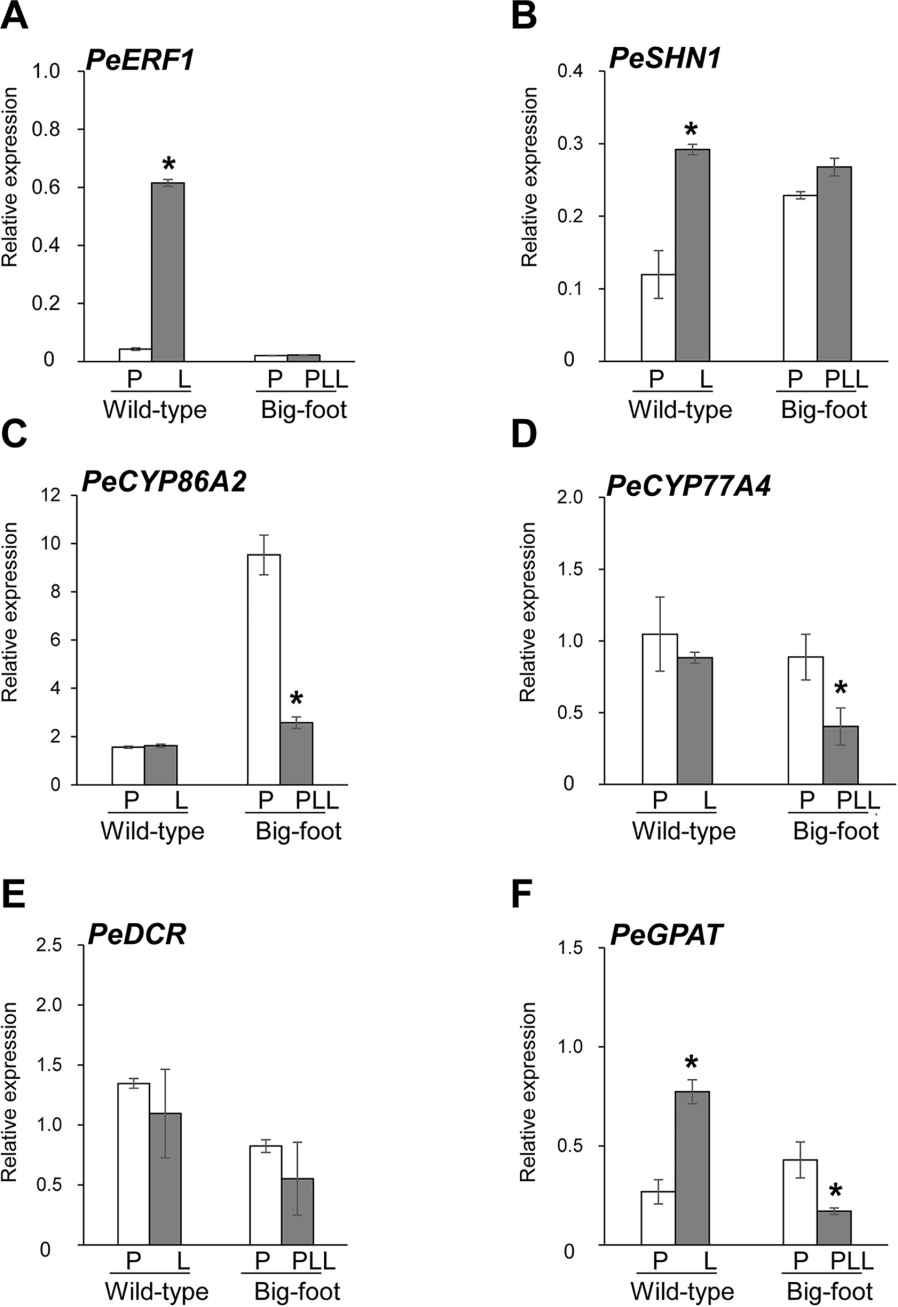
Expression patterns of *PeERF1*, *PeSHN1*, cutin-biosynthetic genes in petal and lip of “wild-type” and “big foot mutant” flowers of *P.* hybrid “Join Big foot TH365.” Transcript level of genes related to lip epidermis development *PeERF1* **(A)**, *PeSHN1* **(B)**, and cutin metabolism-related genes (*PeCYP86A2*, *PeCYP77A4*, *PeGPAT*, and *PeDCR*) **(C**–**F)** in flowers of “wild-type” and “big foot mutant” of *P.* “Join Big foot TH365” were examined. P, L, and PPL were represented as petal, lip, and petal-like lip, respectively. mRNA of petal and lip were extracted from the 2^nd^ floral bud in three independent plants of big foot mutant and wild type. Three technical repeats were performed for each sample. Data are mean ± SD. Numbers above the bars are expression levels after normalization with internal control (*PeActin4*). *P < 0.05 by one-tailed t-test.

To further understand the molecular mechanisms of petal-like lip formation, we investigated gene expression of cutin biosynthesis genes *PeERF1*, *PeSHN1* (*PeCYP86A2*, *PeCYP77A4*, *PeDCR*, and *PeGPAT*) (**Figure 8**), as well as expression of floral morphogenesis genes (B-class [*DEFICIENS* (*DEF*)/*APETALA3* (*AP3*)-like (*PeMADS2-5*) and GLOBOSA (GLO)/PISTILLATA (PI)-like (*PeMADS6*)], *AGAMOUS-LIKE6a* (*PeAGL6a*), and E-class [*SEPALLATA1-4* (*PeSEP1-4*)] MADS box genes) (**Supplementary Figure S10**).

Expression of *PeERF1* was significantly reduced in the lip of the big-foot variant as compared to the wild type. In addition, the expressions of *PeCYP77A4* and *PeGPAT* were reduced in the big-foot variant lip compared to that of the wild-type plant, while expression levels of *PeCYP86A2* and *PeDCR* were not significantly different between the big-foot variant and the wild type. Interestingly, expression of *PeSHN1* was nearly unaffected for petal-like lip in big-foot variant as compared to that of lip in wild-type plant. These results suggest that *PeERF1*, *PeCYP77A4*, and *PeGPAT* were involved in the nanoridge formation in the orchid lip.

For MADS box genes, highest expressions of *PeMADS3, 4 and 6*, and *PeAGL6a* were detected in the lip of “wild-type” flowers, whereas *PeMADS2* expressed higher in the petals (**Figure 8A**). In contrast, only *PeMADS2* and *PeMADS3* expressed higher in petal-like lip compared to the petal in the big-foot variant (**Supplementary Figure S10**). Intriguingly, we found little or no difference in transcriptional levels of *PeMADS4*~*6* between the petal-like lip and the petals of the big-foot variant (**Supplementary Figure S10**).

### Ectopic Expression of *PeERF1* in *Arabidopsis* Resulted in a Typical SHINE Phenotype

**Figure 9:**
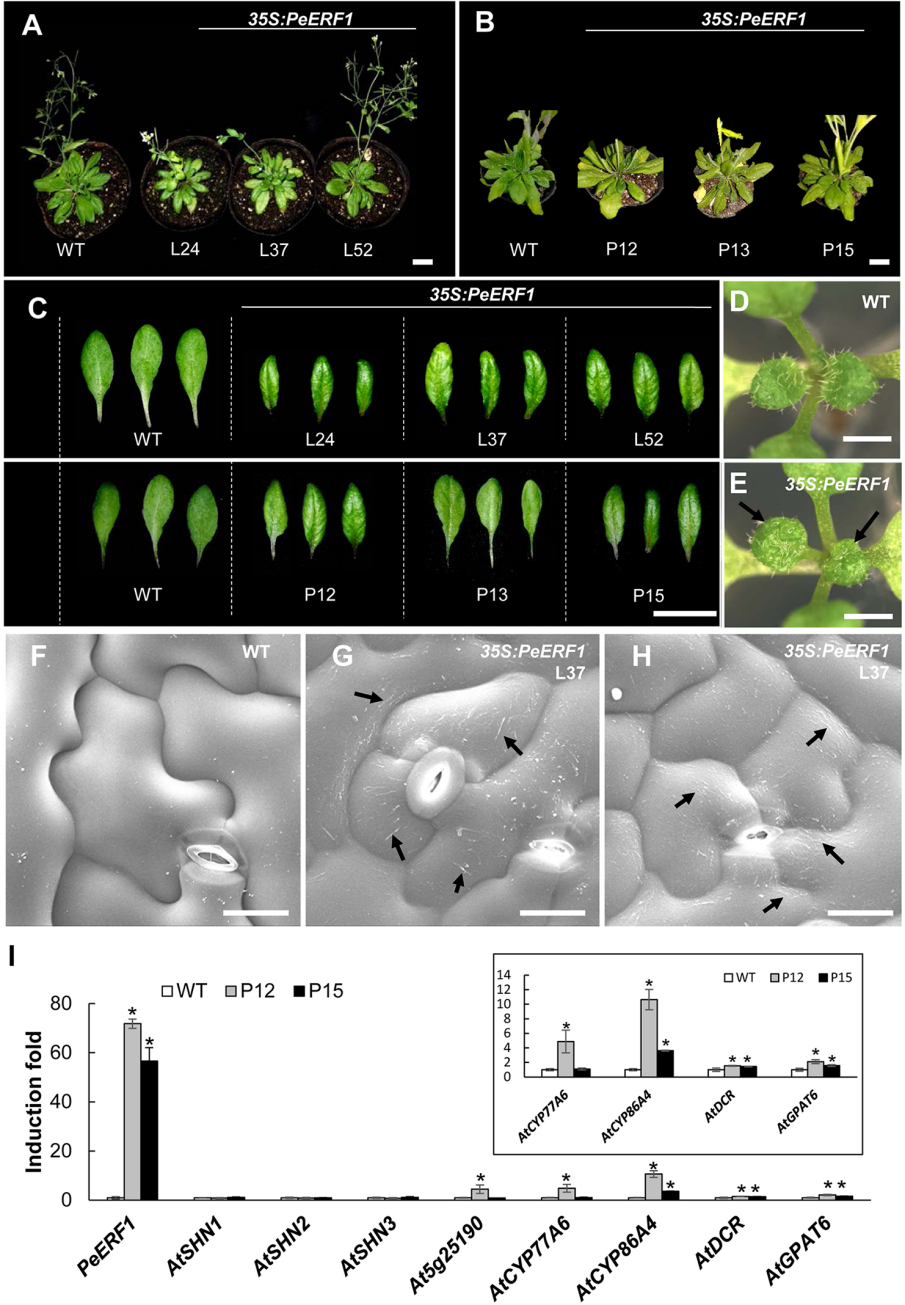
Phenotype analysis and expression patterns of transgenic *Arabidopsis* plants ectopically expressing *PeERF1*. **(A)** Wild-type and *35S:PeERF1* transgenic lines (the first batch, L24, L37, and L52) at 45 days old, and **(B)** wild-type and *35S:PeERF1* transgenic lines (the second batch, P12, P13, and P15) at 60 days old. Scale bars, 5 cm. **(C)** Shiny and curved-down edges of the rosette leaves of 45-day-old wild-type and *35S:PeERF1* transgenic plants of L24, L37 and L52, and 60-day-old wild-type and *35S:PeERF1* transgenic plants of P12, P13, and P15. Scale bars, 2 cm. **(D**–**E)** Seedlings of wild-type and *35S:PeERF1* transgenic plants. Black arrow indicates the top view of the second pair of true leaves with a shiny surface. **(F**–**H)** Micrograph images of adaxial surface of rosette leaf epidermal cells of wild-type and *35S:PeERF1* transgenic line 37. Black arrow indicates ectopic wax deposition in the AF and anticlines of the cell. Scale bars, 10 μm. **(I)** Expression patterns of *PeERF1* and *Arabidopsis* cuticle-associated genes (*AtSHN1-3*, *At5g25190*, *AtCYP86A4*, *AtCYP77A6*, *AtGPAT6*, and *AtDCR6*) in wild type and *35S:PeERF1* overexpressing lines P12 and P15 were determined by qRT-PCR and normalized to the expression level of the *Actin* gene as an internal control. Fold induction of *PeERF1* and 8 genes associated with cuticle biosynthesis in *35S:PeERF1* transgenic plants and wild-type plants, as compared to control. Asterisks were used to indicate statistically significant difference compared with wild-type plants. Three technical replicates were performed for each overexpression line and repeated in two different overexpression lines independently. Data are mean ± SD. *P < 0.05 by one-tailed t-test.

To further characterize the biological function of *PeERF1,* we performed ectopic overexpression of *PeERF1* under the control of CaMV 35S promoter by *Agrobacterium*-mediated transformation in *Arabidopsis*. Two batches of overexpression were performed. In the first batch, a total of 60 T1 transgenic lines were generated, and in the second batch of overexpression, a total of 48 transgenic lines were generated. Most of overexpressing lines showed SHINE phenotypes. Three overexpressing lines (L24, L37, and L52) from the first batch and three overexpression lines (P12, P13, and P15) from the second batch were used for further analysis (**Figure 9**). These plants have enhanced brilliant, shiny green color and curved-down edges rosette leaves as compared to wild-type plants (**Figures 9A–C**). For seedlings of *35S:PeERF1* transgenic lines, the adaxial surface of the second pair of true leaves show a shiny surface with few trichomes as compared with the wild type (**Figures 9D**, **E**). At the cellular level, the adaxial surfaces of rosette leaf epidermal cells of *35S:PeERF1* transgenic plants showed ectopic nanoridge formation and wax deposition on the surface, in contrast to the wild-type leaf with a smooth surface (**Figures 9F–H**, black arrow).

In addition, qRT-PCR was performed to examine the gene expression in the two transgenic lines with obvious phenotype, P12 and P15 from the second batch of overexpression. Ectopic expression of *PeERF1* was accompanied by upregulation of cuticle biosynthesis genes, including *AtCYP86A4*, *AtDCR*, and *AtGPAT6* in two independent lines (**Figure 9I**). Furthermore, the ectopic overexpression of *PeERF1* in *Arabidopsis* did not disturb the expression of the three endogenous *AtSHNs* (*AtSHN1~AtSHN3*) as well as the *PeERF1* orthologous gene *At5g25190* in all ectopic overexpression lines except the expression of *At5g25190* in the line P12 (**Figure 9I**). These results suggest that *PeERF1*, with similar SHINE phenotypes, might be assigned to one of the SHINE-like TFs and can be ectopically overexpressed and functionally involved in leaf cuticle development in *Arabidopsis.*

## Discussion

### PeERF1 Exhibits Partial SHINE Functions but Contains Incomplete SHINE Domains

ERF-Va2 subgroup genes are not known to have a typical SHINE phenotype; rather, they are involved in various developmental and physiological processes (Aharoni et al., 2004; Li et al., 2007; Marques et al., 2013; Trupiano et al., 2013; Nakano et al., 2014; Leida et al., 2016). Recently, berry VvERF045 SHINE domains were found to be involved in berry ripening and epidermal cuticle development. Transgenic grapevine lines overexpressing *VviERF045* show stunted growth, discolored, and smaller leaves, with reduced gene expression for epidermal wax decoration and wax biosynthesis. This indicates that VviERF045 is a potential repressor in epidermis patterning and cuticle development (Leida et al., 2016).

In this study, *PeERF1*_*AP2*-silenced plants showed significantly reduced expressions of *PeERF1* and *PeSHN1* with loose and uneven nanoridges on the lip epidermal surface, accompanied by drastically reduced expression of cutin biosynthesis genes including *PeCYP86A2*, *PeCYP77A4*, and *PeDCR*. Expression of *PeGPAT*, on the other hand, was affected only to a less extent (**Figure 6**). These results suggest that both *PeERF1* and *PeSHN1* are important for cuticle formation. Yet the expression level of *PeSHN1* was much lower than that of *PeERF1*. Similar to the reduced expression of *PeERF1* in the *PeERF1_SHN*-silenced plants, the expressions of *PeCYP86A2*, *PeDCR*, and *PeCYP77A4* were reduced significantly in *PeERF1_AP2*-silenced plants. The expression of *PeCYP77A4* was not affected in the *PeERF1_SHN*-silenced plants (**Figure 6D**). The expression of *PeGPAT* was reduced in both *PeERF1_AP2*-silenced and *PeERF1-SHN* plants, but to a lesser extent (**Figure 6F**). Therefore, it appears that *PeCYP77A4* and *PeGPAT* are regulated by PeERF1 and/or PeSHN1 and downregulation of these genes might explain the cuticle formation defects in the *PeERF1*-silenced plants. Based on our observations, it is also suggested that proper expression of *PeCYP77A4* and *PeGPAT* might involve additional TF(s). Thus, our results suggest that *PeERF1* as a SHN-like homolog is a potential activator in the lip epidermal cell patterning of *Phalaenopsis* flowers. Furthermore, transgenic *Arabidopsis* lines overexpressing *PeERF1* showed typical SHINE phenotypes similar to *AtSHN*-overexpressing *Arabidopsis* (**Figures 9A–C**). The *PeERF1* overexpressing plants enhanced the expression of *AtCYP86A4*, *AtDCR*, and *AtGPAT6* without disturbance of the expression of endogenous *AtSHNs* and the homologous gene *At5g25190* except the expression of *At5g25190* in the line P12 was increased (**Figure 9I**). With the fact that *At5g25190* does not have the function for cuticle formation (Aharoni et al., 2004), the phenotype observed in the overexpressor P12 was due to the *PeERF1 per se*. Therefore, our results suggest that *PeERF1*, as a *SHINE*-like TF, increase the *Arabidopsis* cuticle-associated genes and result the shiny surface of rosette leaves.

Recent studies have indicated the importance of CMV-1 and CMV-2 motifs of SHINE domains. A highly conserved valine (V) residue in the complete CMV-1 motif in barley has been found to be associated with lipid biosynthesis of grain (Taketa et al., 2008). When V is changed to aspartic acid (D) in the complete CMV-1 motif, instead of the typical hulled caryopsis in barley, a naked one results. This is associated with lipid formation of caryopsis and hull adhesion (Taketa et al., 2008). In addition, the existence of a C-terminal 30-amino-acid region of the CMV-2 motif is also important for transcriptional activation of SlERF52 (Nakano et al., 2014). The protein sequences of PeERF1 were more distinguished from other members of the ERF-Va2 subgroup (**Figure 4**). We found that PeERF1 has a serine (S) residue at position 5 of the incomplete CMV-2 motif, whereas several members of the Va2 subgroup have a histidine (H) residue at the same position (**Figure 4B**).

### PeERF1, PeSHN1, and Other TFs Together Regulate Cutin Biosynthesis Genes for Nanoridge Formation

Studies of SHINE genes modulating cuticle permeability and epidermal cell patterning have revealed the requirement of the downstream synthesis of cutin polyesters (Kannangara et al., 2007; Li-Beisson et al., 2009; Panikashvili et al., 2009; Shi et al., 2011; Shi et al., 2013; Petit et al., 2016; Mazurek et al., 2017). A recent model was formulated of the association between cutin biosynthesis and nanoridge formation of the petal cuticle (Mazurek et al., 2017). However, although several SHN putative downstream target genes related to cuticle formation have been reported, SHN TFs do not bind directly to most of their presumed targets. In fact, they require an interacting partner for SHN-mediated target regulation (Kannangara et al., 2007). So far, only the promoter regions of CYP86A cytochrome P450s (AtCYP86A4, AtCYP86A7, and SlCYP86A69) and GSDL-motif lipases (AtRXF26) have been found to be activated by SHNs in *Arabidopsis* and tomato (Shi et al., 2011; Shi et al., 2013).

When *PeERF1* was silenced, we found associated downregulation of cutin biosynthesis gene expressions and reduced numbers of nanoridges on lip abaxial and adaxial surfaces. However, even though the expressions of *PeCYP86A2* and *PeDCR* were significantly reduced, that of *PeGPAT* was only mildly reduced in lip epidermis in both *PeERF1*-silenced plants (**Figure 6**). Thus, *PeCYP86A2* and *PeDCR* may be downstream genes of *PeSHN1*, and PeCYP77A4 a downstream gene of *PeERF1*. *PeGPAT* was regulated by *PeERF1*, *PeSHN1*, and non-SHN like TFs. Hence, *PeERF1* as a SHN-like TF is involved in decorating the floral organ epidermal surface by regulating downstream cutin biosynthesis genes.

### *PeERF1*, as a Downstream Target of Floral Morphogenesis Genes, is Involved in the Late Stage of Lip Morphogenesis

Recently, the roles of B- and E-class MADS-box genes were revealed for orchid tepal development (Tsai et al., 2004; Tsai et al., 2005; Lu et al., 2007; Hsieh et al., 2013a; Su et al., 2013b; Pan et al., 2014; Hsu et al., 2015b; Huang et al., 2015; Huang et al., 2016). We have shown that knock-down expression of E-class *PeSEP1-4* genes reduced the expression of *PeERF1*. *PeSEP1-*silenced plants had a changed ultrastructure in terms of nanoridge formation, of the floral epidermal cells (Pan et al., 2014). This result suggests that *PeERF1* could be a downstream gene of E-class MADS box genes.

In this study, we found that the expression of *PeERF1* was reduced to almost zero in the big-foot somaclonal variants with a petal-like lip, along with the loss of gene expression of B-class (*PeMADS4*, *PeMADS5*, and *PeMADS6*) and E-class (*PeAGL6a*) MADS box genes (**Supplementary Figure S10**). These results suggest that the developmental program of lip morphogenesis is very complex, and *PeERF1* is a downstream target gene of B- and E-class MADS box genes and is involved in lip morphogenesis.

### The Timing for VIGS Phenotype and Off Target VIGS

Our previous VIGS results of MADS box silencing data showed the highest silencing efficiency at 4–7 weeks post-silencing with the first four flowers blooming after viral inoculation (Hsieh et al., 2013a; Hsieh et al., 2013b; Pan et al., 2014; Hsu et al., 2015a). This indicates that MADS box genes are induced in early floral morphogenesis.

*PeSHN1* as an SHN ortholog contained the complete SHINE domains and was assigned to the ERF-Va1 subgroup (SHINE clade) (**Figure 4**). However, *PeERF1* was expressed at late stage lip morphogenesis so that VIGS phenotype was more distinct on the late emerged floral buds. The identity of *PeSHN1* with *PeERF1*_AP2-silenced and *PeERF1*_SHN-silenced regions were 71.8% and 47.1%, respectively. Yet, no contiguous matches were longer than 11 nt for the *PeSHN1* coding region and the VIGS fragment (**Supplementary Figure S7**). A nucleotide identity of less than 11 nt on target mRNA has previously been shown to reduce the chances of silencing induction (Senthil-Kumar and Mysore, 2011; Hsieh et al., 2013a). The off-target effects of *PeSHN1* silencing that occurred in all *PeERF1*-silenced plants may be due to sequence similarity. The combined effects of specific silencing of *PeERF1* and off-target silencing of *PeSHN1* together resulted in a change of nanoridge formation in lip epidermal cell patterning in the *PeERF1*-silenced plants, yet *PeSHN1* expressed much lower than *PeERF1* in the *Phalaenopsis* orchids.

### Distinct Sculpture on the Lip Epidermal Surface Reveals the Special Deceit Pollination Strategy in *Phalaenopsis*

To contribute to successful sexual reproduction in higher plants, the perianth of flower creates various cues, such as tactile, visual, and olfactory signals, to reward or not reward (deceive) pollinators. In Orchidaceae, approximately one-third of orchid species have a deceit pollination strategy by using general floral signals without rewarding pollinators with nectar or pollen (Ackerman, 1986; Nilsson, 1992; Jersáková et al., 2006). Although numerous *Phalaenopsis* species are scentless, with a diversity of colorful perianths, floral morphology, and floral scents, bees are the major pollinators of *Phalaenopsis* flowers *via* food-deceptive pollination (Roman Kaiser, 1993; Hsiao et al., 2006; Tsai et al., 2008).

In this study, we examined the floral epidermal cell surfaces of *Phalaenopsis* species (**Figure 1**; **Supplementary Figure S1**); the tissues of sepals and petals had a papillae shape with a protuberance on the smooth epidermal surface, whereas the lip harbored numerous nanoridges on the epidermal surface. The biological function of nanoridges on the floral organs for pollinator attraction has been linked with the unique visual and tactile signals they produce (Whitney et al., 2009; Prüm et al., 2011; Rands et al., 2011; Whitney et al., 2011b; Prüm et al., 2012; Kourounioti et al., 2013; Prüm et al., 2013; Adachi et al., 2015; Moyroud et al., 2017).

We propose three steps of a special deceit pollination strategy in the scentless *Phalaenopsis* species. First, the papillae cell shape on the sepal and petal epidermis creates big brilliant visual cues to attract pollinators from a distance. Then, the convex cell shape with heavy nanoridges on the top of the lip epidermal cells generates more “flashy” visual cues (similar to guide lights on an aircraft runway) and direct pollinators to land on the lip instead of sepals and petals. Finally, the distinctive split lip with central lobes and lateral lobes creates a tunnel-like structure, and the nanoridges on the lip epidermis generate tactile cues that help pollinators walk and explore the area. During this process, because of the heavy and dense nanoridges on the top of the lip epidermis, the pollinators may slip toward the column and attach to the pollinia. While visiting subsequent flowers, successful pollination may occur when attached pollinia are placed into the stigmatic cavity underneath the column. The slippery quality of nanoridges for beetles has been reported (Prüm et al., 2011), and the slip-and-fall pollination mechanism related to the ultrastructural characterization of the floral lip was also shown for *Gongora bufonia* (Orchidaceae) (Adachi et al., 2015).

## Conclusion

In conclusion, our results demonstrate that PeERF1, as an SHN-like TF, was involved in lip epidermal cell morphological formation at the last flowering stage by regulating lip nanoridge development in *Phalaenopsis* flowers. In addition, the heavy nanoridges on the lip epidermis, as typical lip features, may be essential for the pollination mechanism of *Phalaenopsis.* This study gives a better understanding of the transcriptional regulation of the late stage development of lip morphogenesis and the special pollination mechanism of *Phalaenopsis.*

## Data Availability Statement

The datasets generated for this study can be found in the PeERF1, MG948436; PeSHN1, XM_020736987.1; PeCYP86A2, XM_020732683.1; PeCYP77A4, XM_020725159.1; PeGPAT, XM_020727266.1; PeDCR, XM_020725429.1.

## Author Contributions

P-HL, W-HC, and H-HC conceived the research plans. P-HL performed most of the experiments, analyzed the data, and wrote the article with contributions from all the authors. L-MH assisted in the identification and expression analysis of cutin biosynthesis genes. Z-JP assisted with the performance of VIGS experiments. W-NJ and M-CC performed Cryo-SEM analysis and provided service for Cryo-scanning electron microscope.

## Funding

This work was supported by grant no. MOST-107-2313-B-006-003-MY3 from the Ministry of Science and Technology, Taiwan.

## Conflict of Interest

The authors declare that the research was conducted in the absence of any commercial or financial relationships that could be construed as a potential conflict of interest.

## Abbreviations

AF, anticlinal field; CF, central field; Cryo-SEM, Cryo-scanning electron microscopy; ERF, ethylene responsive factor; RT-PCR, reverse transcription–polymerase chain reaction; SHN/WIN, SHINE/WAX INDUCER; TFs, transcription factors; VIGS, virus-induced gene silencing.
