# Supplementary material for: PeERF1, a SHINE-Like Transcription Factor, Is Involved in Nanoridge Development on Lip Epidermis of Phalaenopsis Flowers
Source: Front Plant Sci. 2020 Jan 30;10:1709. doi: 10.3389/fpls.2019.01709 (PMC7002429; doi:10.3389/fpls.2019.01709)
Supplement: Supplementary file 1 [file DataSheet_1.pdf]

Supplemental Table

Supplemental Table 1. List of primers used in this study

Primer name	Sequence (5'→3')
**3' and 5' RACE primers**	
PeERF1-3-F1	GCAGAGATACAGAGGAGTGAGGCAGAG
PeERF1-3-F2	GCTGGAGAGATGCTGCATTGCTTC
PeERF1-5-R1	CCAATAGAGAATGAGGAGTCATGCAGC
PeERF1-5-R2	CGAAATAAAGCCCCAGGCATAATGC
**RT-PCR analyses primers**	
PeERF1-RT-F1	GATGCTGCATTGCTTCTCAAGAG
PeERF1-RT-R1	TGAGGAGTCATGCAGCTGACC
CymMV-CP-F	GAAATAATCATGGGAGAGCC
CymMV-CP-R	AGTTTGGCGTTATTCAGTAGG
ORSV-CP-F	ACGCACAATCTGATCCGTA
ORSV-CP-R	ATCCGCAGTGAAAACCC
**Real time RT-PCR analyses primers**	
PeERF1-qF1	ATTATGCCTGGGGCTTTATTTCGGAAAC
PeERF1-qR1	AGCTGACCGGTTAATGATGATGATGATG
PeSHN1-qF1	ACATTGGTGTCTGGCAGAAGCG
PeSHN1-qR1	AAGGCGATGGCAGAGGCATG
PeCYP86A2-qF1	GGCAGGCTCCGCTATCACTTACT
PeCYP86A2-qR1	TGAAGAAATCGGCTGGCGGTTC
PeCYP77A4-qF1	GGTTACGACATTCCGGCGGA
PeCYP77A4-qR1	GGCAAAAACCTCTCCGGCTT
PeDCR-qF1	TACTTCGGCAACCTCATCCAAGC
PeDCR-qR1	TCGATGGCCTTCTGCAGTAGTCC
PeGPAT-qF1	TTGGATAGCACCAAGTGGTGGAAG
PeGPAT-qR1	GCCAGAATGCTCGGACAATCTTCT
PeActin4-F	TTGTGAGCAACTGGGATGACAT
PeActin4-R	GCCACGCGAAGTTCATTGT
AtSHN1-qF1	AACCGATATTTCAGCTTCGTCCAC
AtSHN1-qR1	TGAGGGATGGGGAAGGAGACTT
AtSHN2-qF1	TCAGAGGAAGGCTCCGATCAC
AtSHN2-qR1	CGTTAGGTCTTTGCAGCTCTTCCTT
AtSHN3-qF1	CATCAAATCGAACGGTTCAAATTCC
AtSHN3-qR1	CGGTGTCTGGTCTTTACAGTTCTTC
AT5G25190-qF1	CAACCCTAATGCCATTCCTACTTCG

AT5G25190-qR1	GCGTTTGCTTGGTCATTTGAAGAG
AtCYP86A4-qF1	ATGAAGTCGACTTGGGGAGAAGA
AtCYP86A4-qR1	GCCACGAACCGATATTGATCG
AtCYP77A6-qF1	CCTCCGACATACTTTACGTTGACTC
AtCYP77A6-qR1	GATCCTCATTTATTCCCGGAAGATA
AtDCR-qF1	GCACGTAACGACGAGTGGGAGA
AtDCR-qR1	CACGAAACCTCGGTGAGCTTCC
AtGPAT6-qF1	TCGTGATATGTCCCGAGGGAA
AtGPAT6-qR1	GTGTTGATCGCCACGGGAAC
AtActin1-qF1	GGCGATGAAGCTCAATCCAAACG
AtActin1-qR1	GGTCACGACCAGCAAGATCAAGACG
**VIGS experiment primers**	
PeERF1-ap2-F1	GGGGACAAGTTTGTACAAAAAAGCAGGC TAGTGAGGCAGAGGCACTGG
PeERF1-ap2-R1	GGGGACCACTTTGTACAAGAAAGCTGGGT TCGCTCCGCACATGAACTTC
PeERF1-shn-F1	GGGGACAAGTTTGTACAAAAAAGCAGGC TTAAGCTGGAGAGATGCTGC
PeERF1-shn-R1	GGGGACCACTTTGTACAAGAAAGCTGGGT TGGCAGTAGAAGAAGATG
**Transformation in *Arabidopsis* primers**	
PeERF1-OE-F1	CCCGGGACGCACACAATAGCAGAATGCCAAAACTG
PeERF1-OE-R1	CCCGGGACAATAGAGAATGAGGAGTCATGCAGCTG

Supplemental Figure legends

Supplemental Figure S1. Ultrastructure of floral epidermal cells in *Phalaenopsis* flowers.

(A) Floral morphology of *P. aphrodite* subsp. *formosana*. Scale bars, 5 cm. Abbreviations: Se, sepal; Pe, petal; Li, lip; Co, column. (B-D) Cyro-SEM epidermal cells of perianth organs of *P. aphrodite* subsp. *formosan*. White arrows indicated a protuberance on the top of the cell. Black dashed arrows indicated irregular nanoridges in the CF of the cell. White arrowheads indicated parallel nanoridges in the AF of the cell. Black arrowheads indicated irregular nanoridges in the AF of the cell. Scale bars, 30 μm.

Supplemental Figure S2. Virus-induced gene silencing (VIGS) of *PeERF1*.

(A) The inserts for gene silencing were in the antisense orientation relative to the CymMV coat protein. "*PeERF1_AP2*-silenced" indicated gene silencing of the 142-nt conserved AP2 domain region (139-281 nt). "*PeERF1_SHN*-silenced" indicated gene silencing of the 175-nt in the incomplete "SHINE domains" (CMV-1 and CMV-2 motifs) (346-521 nt). (B-G) VIGS phenotype of mock-treated, *PeERF1_AP2*-silenced and *PeERF1_SHN*-silenced *P.* OX Red Shoes 'OX1408' plants, respectively. (B-D) The front view of intact mature flowers from the 2nd blooming flowers of mock-treated and *PeERF1*-silenced plants. Scale bars, 1 cm. (E-G) Whole plant of mock-treated and *PeERF1*-silenced plant. Scale bars, 5 cm.

Supplemental Figure S3. Continuously altered distribution of ultrastructural nanoridges on the lip epidermis from 1st flowers to 7th flowers of *PeERF1_AP2*-silenced plants.

(A-AB) Cryo-SEM was used to examine the changes in cellular morphology from the 1th to the 7th blooming flowers of silenced plants (stage 5, floral diameter of 12 cm). Top view of adaxial and abaxial sites of lip central and lateral lobe epidermis from 1st flowers to 7th flowers of *PeERF1_AP2*-silenced plants. More severe phenotypes with looser, uneven and hollowed out distribution of nanoridge structures were exhibited on the lip epidermis of the 6th and 7th flowers. Scale bars, 50 μm.

Supplemental Figure S4. Cell shapes of lip epidermal cells of *PeERF1*-silenced *P.* OX Red Shoes 'OX1408' flowers.

(A-L) Side view of adaxial and abaxial sites of lip central and lateral lobe epidermis in mock-treated, *PeERF1_AP2*-silenced, and *PeERF1_SHN*-silenced plants, respectively. Black arrows indicated node-like nanoridges in the CF of the cell. White dashed arrows indicated parallel nanoridges in the CF of the cell. White arrowheads indicated parallel nanoridges in the AF of the cell. Different cell shape types of floral epidermal cells are in the lower left corner of each panel. Scale bars, 30 μm

Supplemental Figure S5. Ultrastructures and cell shapes of sepals and petals epidermal cells from the 2nd blooming flowers of *PeERF1*-silenced *P.* OX Red Shoes 'OX1408' plants.

(A-X) Top view and side view of adaxial and abaxial sites of sepals and petals epidermis in mock-treated, *PeERF1_AP2*-silenced, and *PeERF1_SHN*-silenced plants, respectively. White arrows indicated a protuberance on the top of the cell. Different cell shape types of floral epidermal cells were in the lower left corner of each panel. Scale bars, 30 μm.

Supplemental Figure S6. Expression patterns of *PeERF1* and cuticle-associated

genes in sepal and petal of *PeERF1*-silenced plants.

Transcript level of *PeERF1* (A), *PeSHN1* (B), and cutin metabolism-related genes (*PeCYP86A2*, *PeCYP77A4*, *PeGPAT*, and *PeDCR*) (C-F) in mock-treated and *PeERF1*-silenced flowers. mRNA of sepal and petal were extracted from the 5th floral bud at 30 d post-inoculation; data are mean ± SD; the same letters above the bars indicate no statistical difference by Duncan's multiple range test ($P < 0.05$).

Supplemental Figure S7. Multiple alignment of regions in *PeERF1* gene used for VIGS experiment.

Red box indicates the regions for primer design to amplify cDNA fragments for *PeERF1_AP2* silenced (A) and *PeERF1_SHN* silenced (B) constructs. The identities of *PeERF1_AP2*-silenced region and *PeERF1_SHN*-silenced region compared to *PeSHN1* were 71.8% and 47.1%, respectively.

Supplemental Figure S8. Ultrastructures and Cell shapes of sepal and petal epidermal cells of somaclonal variants with normal lip or enlarged petal-like lip mutants.

(A-P) Top view and side view of adaxial and abaxial sites of sepal and petal epidermis in "normal type" and "big foot mutant" of somaclonal variants of *P.*'Join Big foot TH365' flowers, respectively. White arrows indicated a protuberance on the top of the cell. Different cell shape types of floral epidermal cells were in the lower left corner of each panel. Scale bars, 30 μm.

Supplemental Figure S9. Ultrastructures and Cell shapes of normal lip and enlarged lip of *P*. 'OX Big foot' **somaclonal variants.**

The lip ultrastructure of abadaxial and adaxial sides of somaclonal variants of *Phal.*

‘OX Big foot’ from top view and side view for central lobe and lateral lob of lip. Scale bars, 30 µm.

Supplemental Figure S10. Expression patterns of floral development-related genes in petal and lip of “normal type” and “big foot mutant” flowers of *P.* ‘Join Big foot TH365’.

Transcript level of genes related to B-class (*PeMADS2–6*) genes (A-E), *PeAGL6a* (F) and E-class (*PeSEP1–4*) MADS box genes (G-J) in flowers of normal type and big-foot mutant of *P.* ‘Join Big foot TH365’ were examined. P, L and PPL were represented as petal, lip and petal-like lip, respectively. mRNA of petal and lip were extracted from the 2th floral bud. Data are mean ± SD. Numbers above the bars are expression levels after normalization with internal control (*PeActin4*). *P < 0.05 by one-tailed t-test.

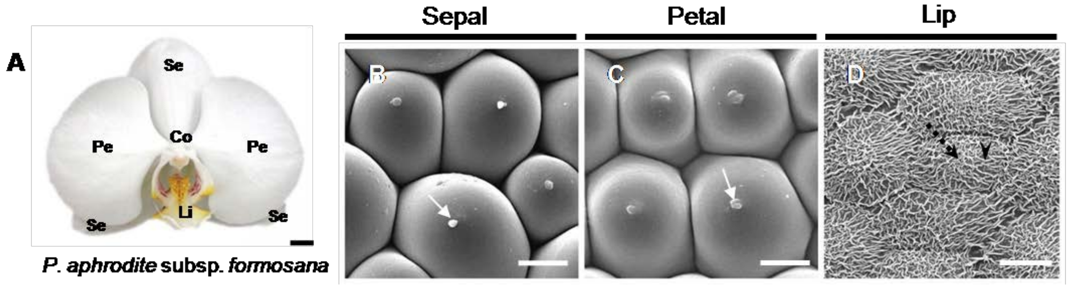

Lai *et al*., Supplementary Figure S1

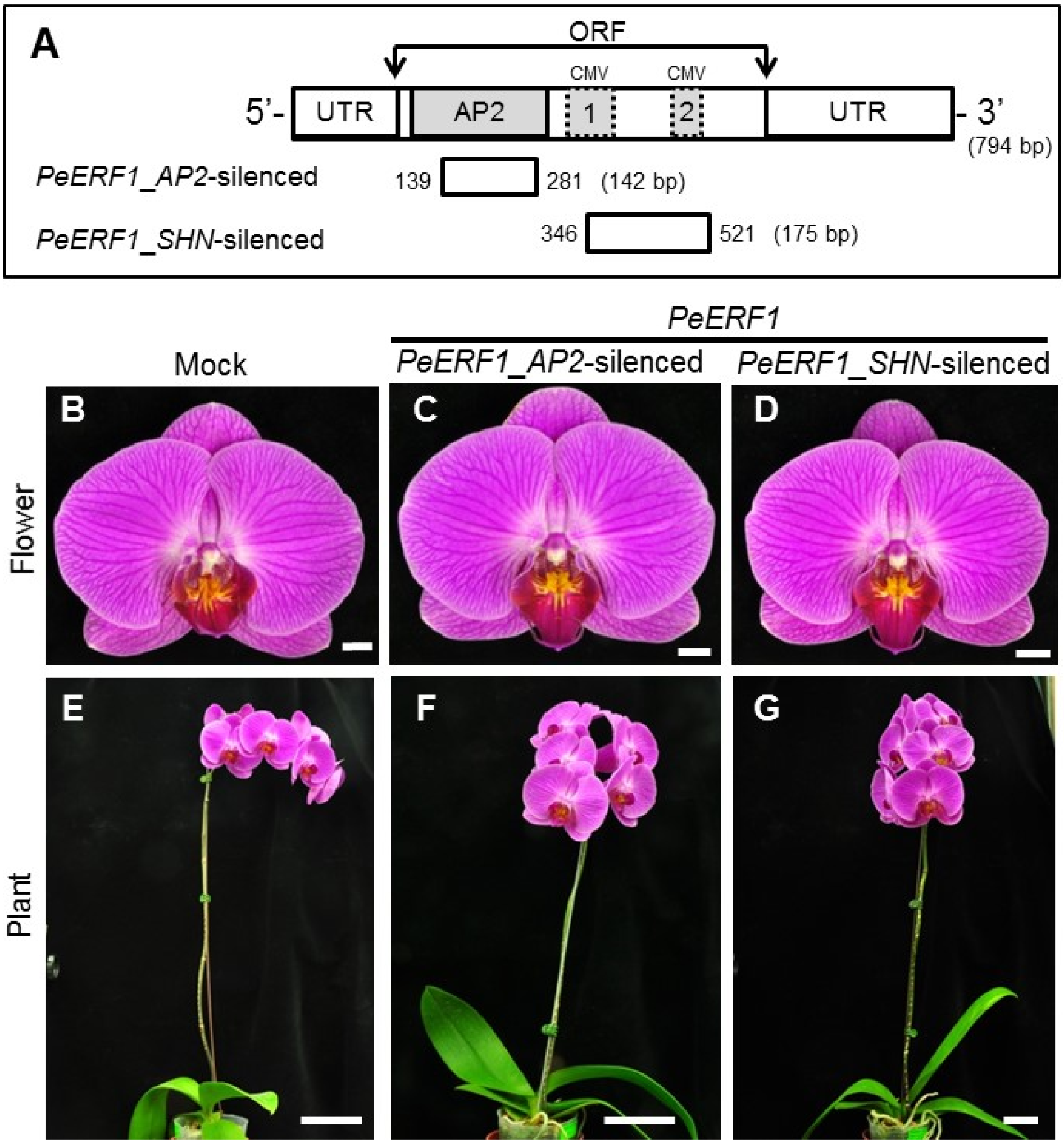

Lai *et al*., Supplementary Figure S2

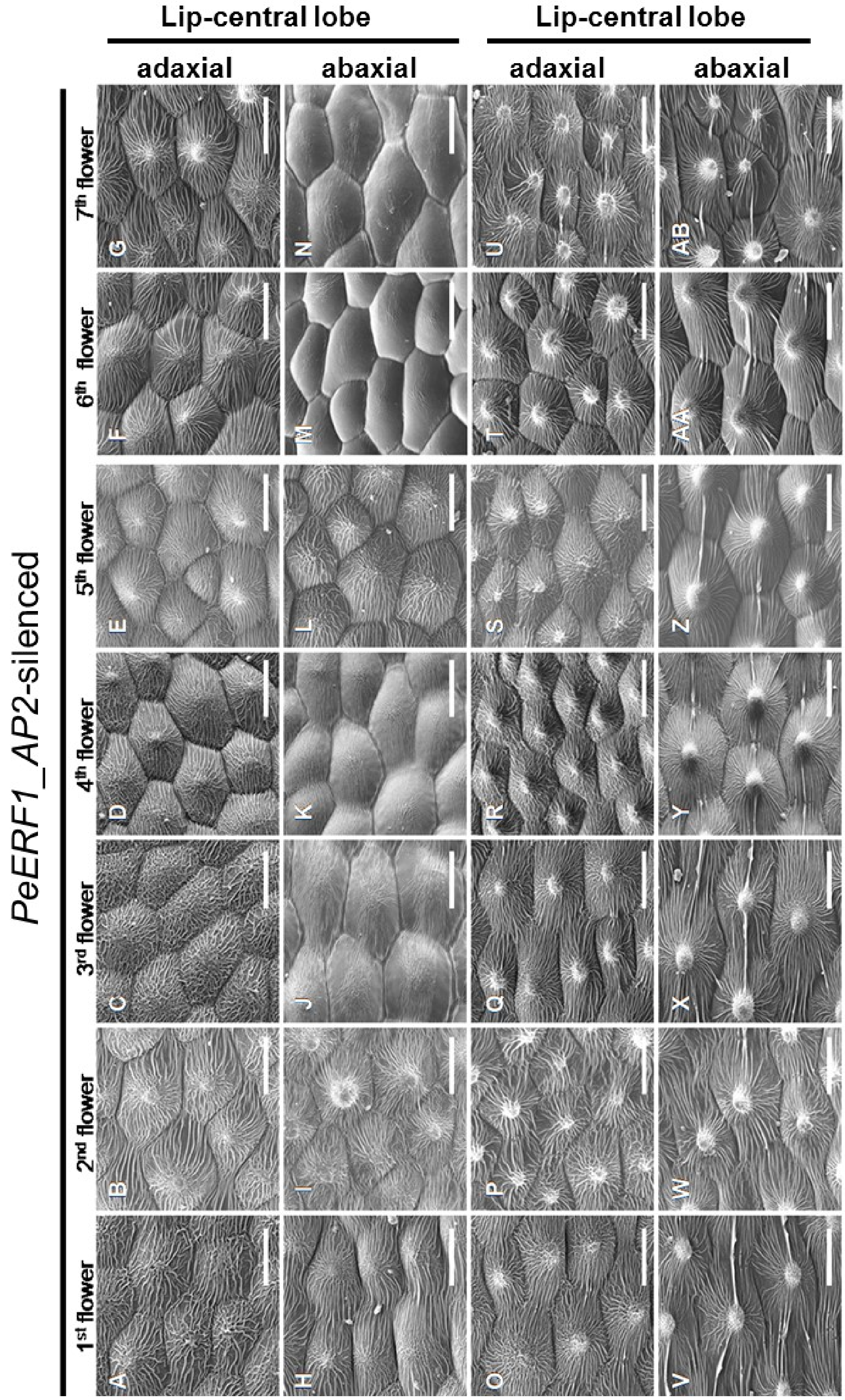

Lai *et al*., Supplementary Figure S3

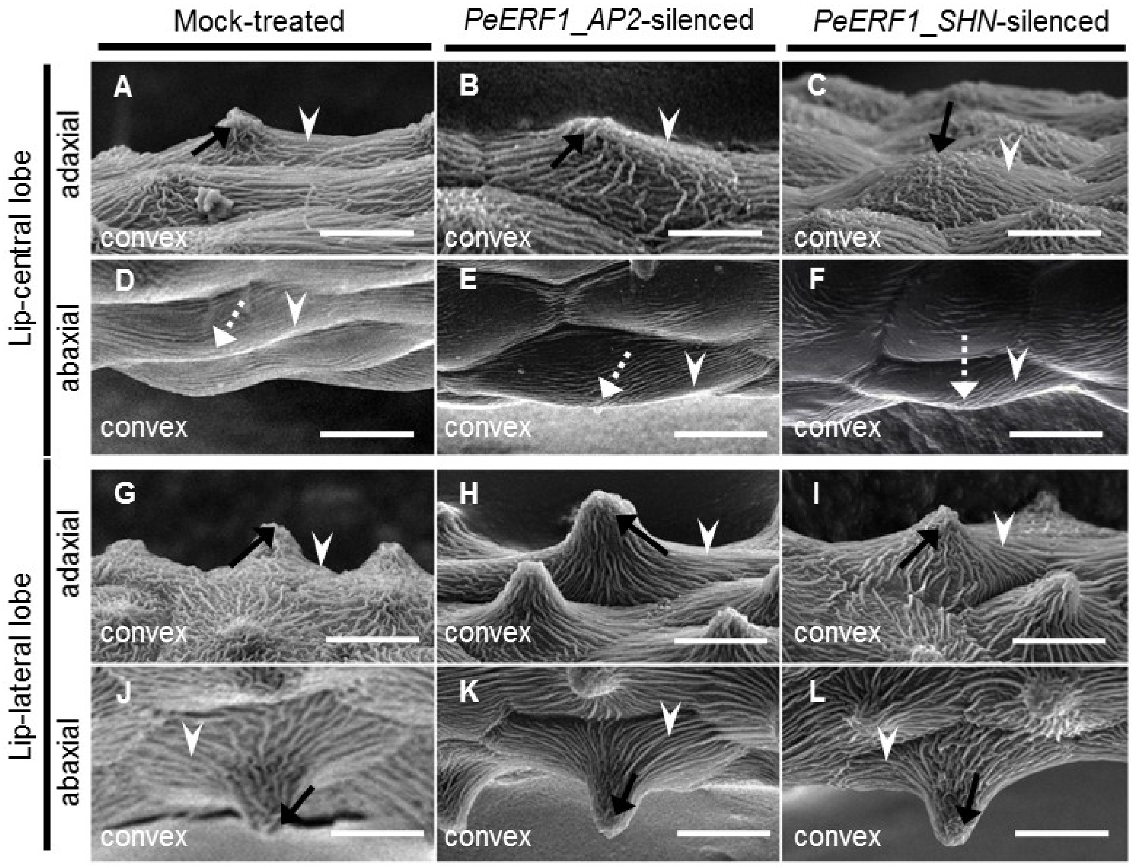

Lai *et al*., Supplementary Figure S4

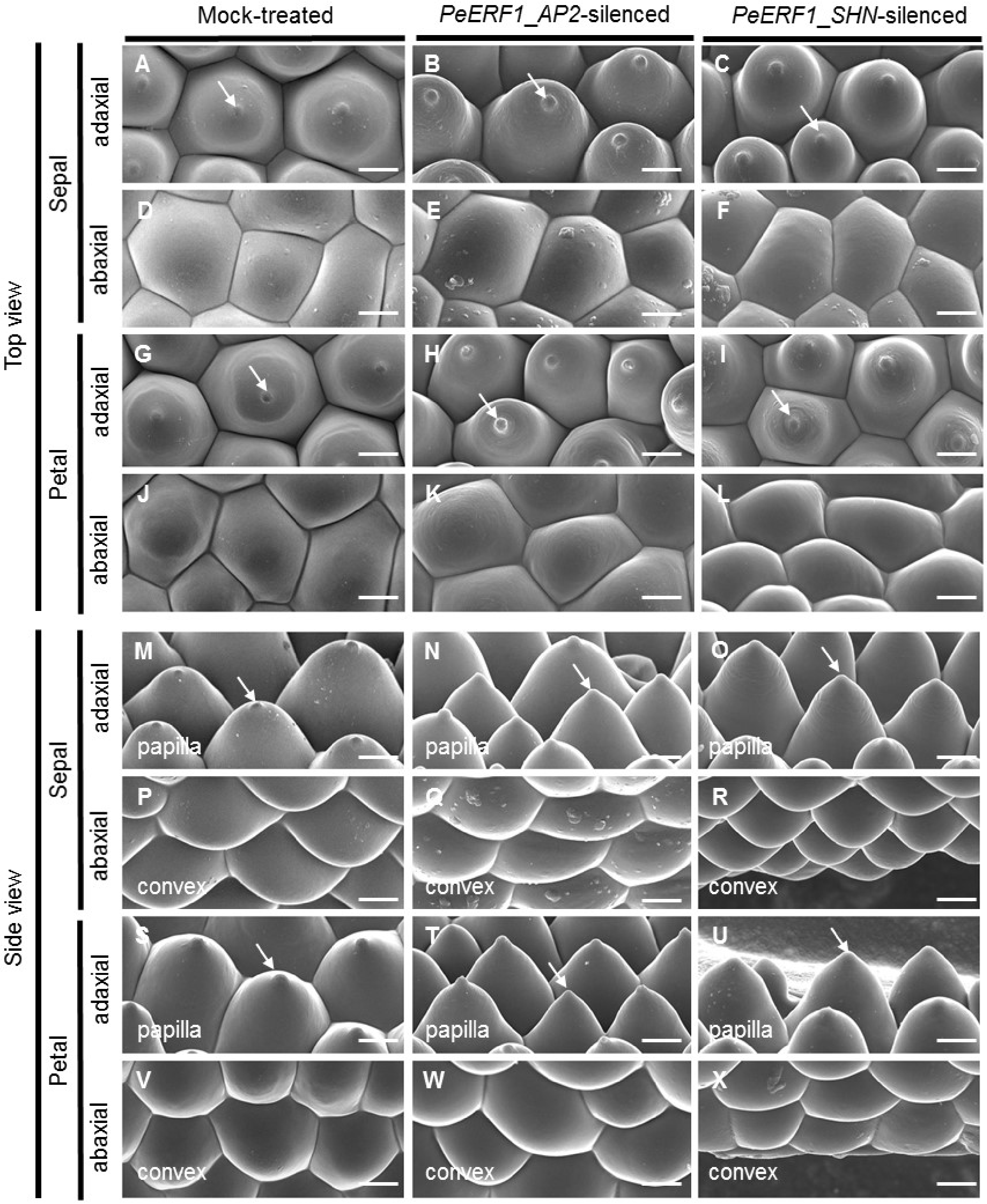

Lai *et al*., Supplementary Figure S5

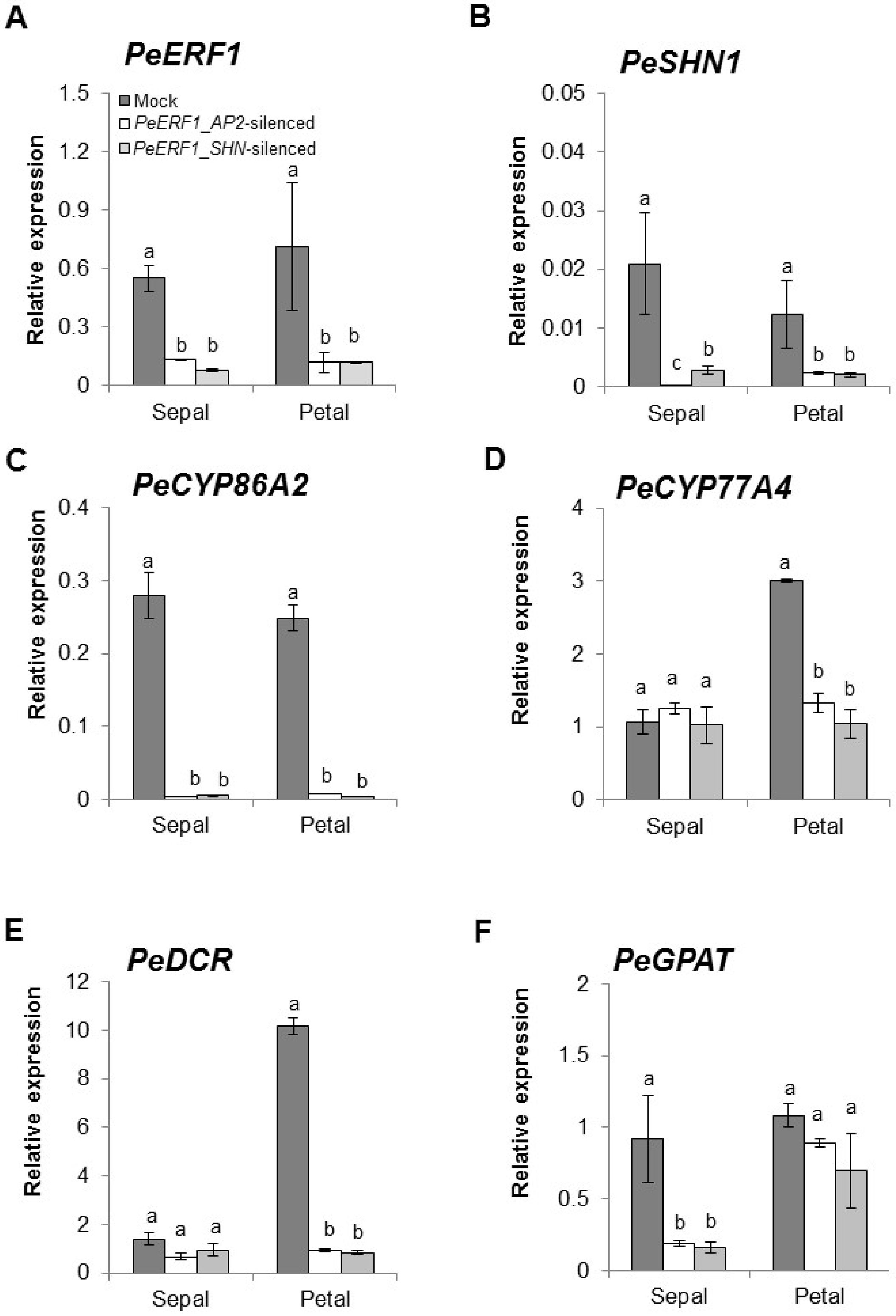

Lai *et al*., Supplementary Figure S6

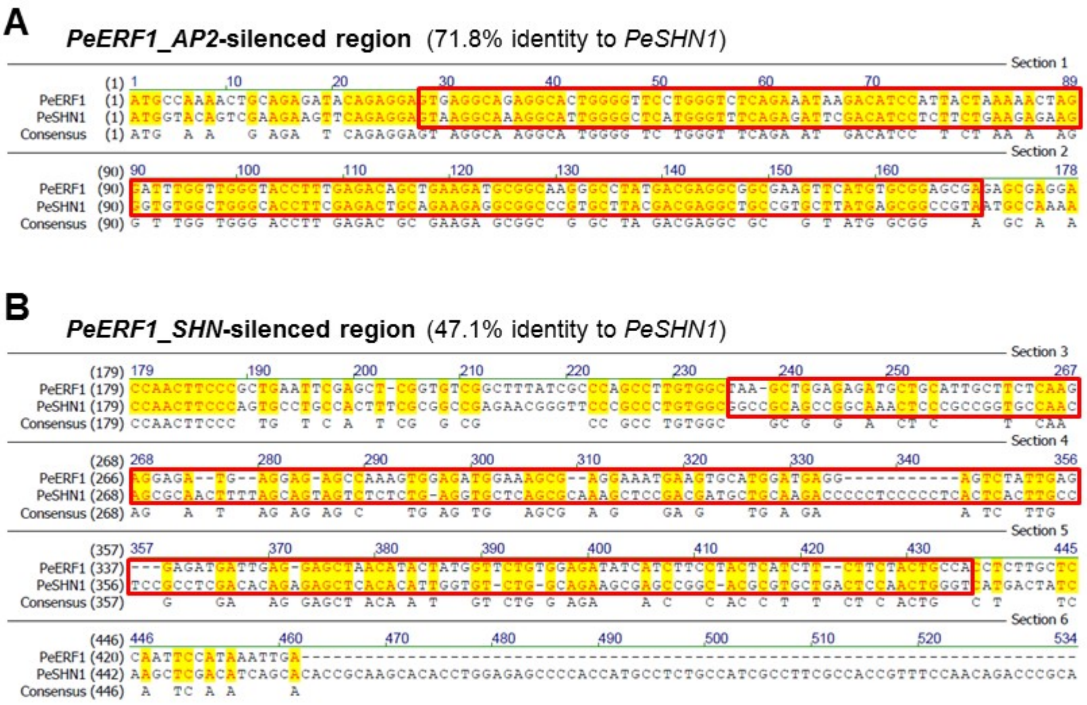

Lai *et al*., Supplementary Figure S7

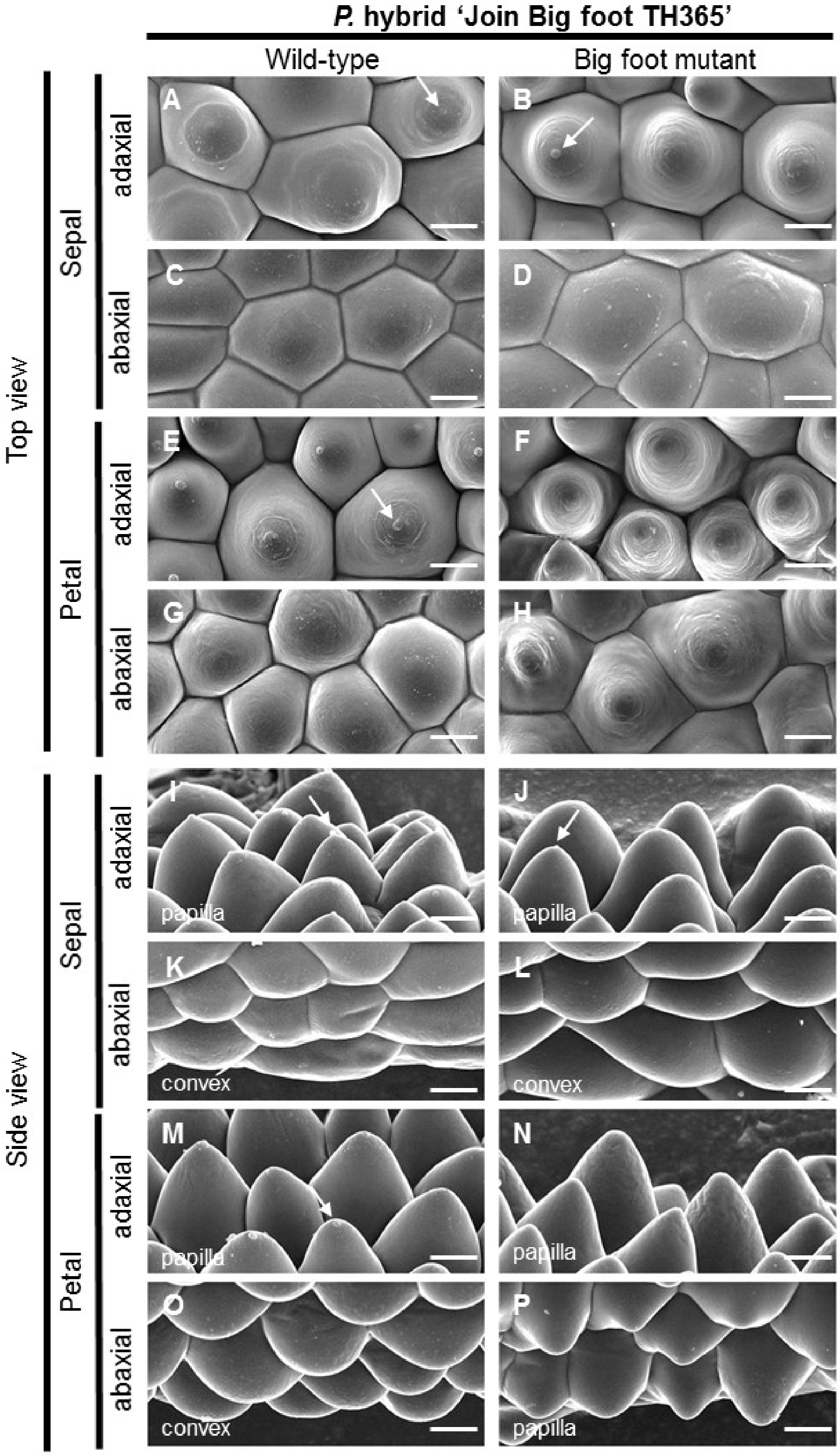

Lai *et al*., Supplementary Figure S8

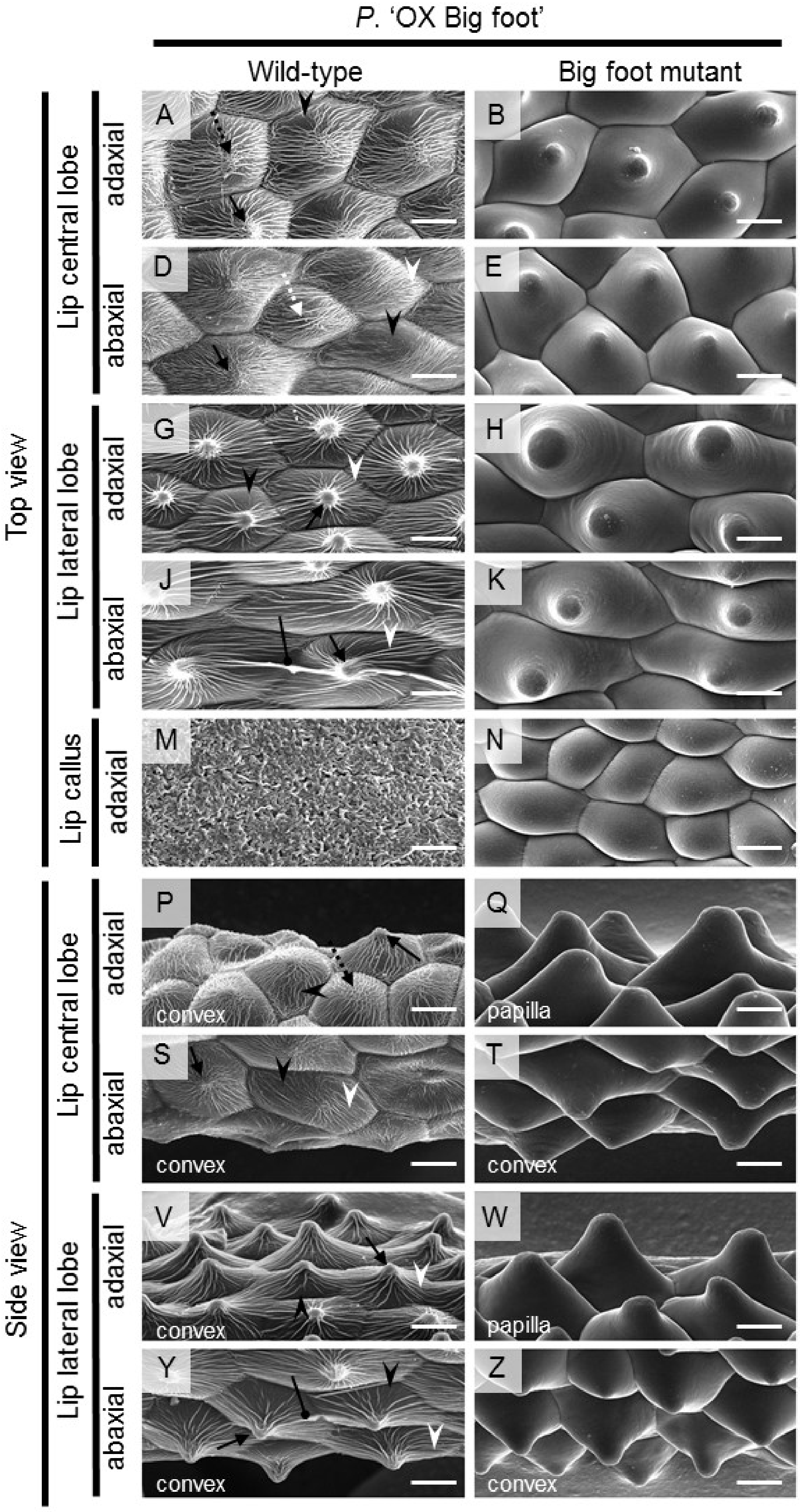

Lai *et al*., Supplementary Figure S9

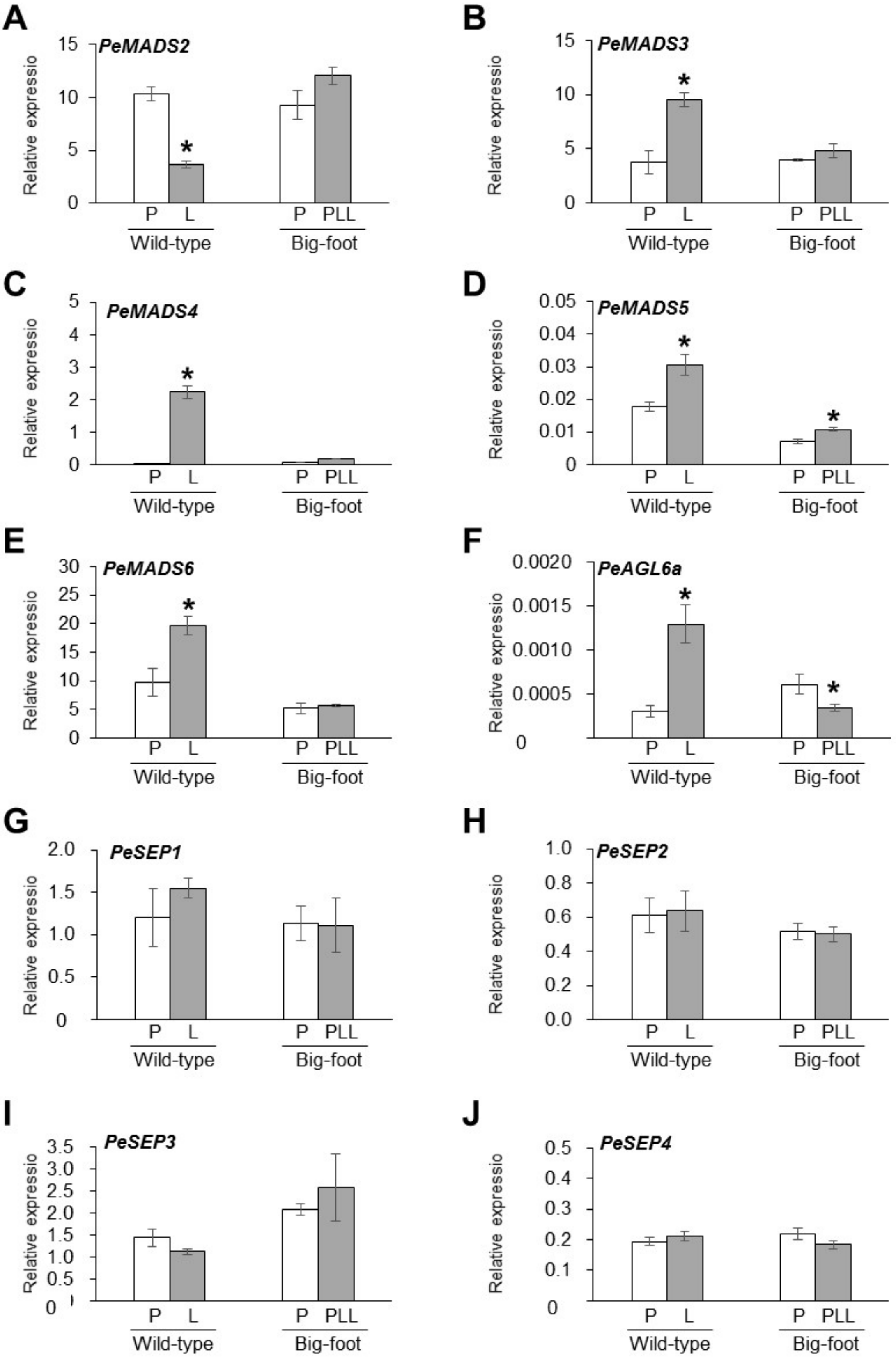

Lai *et al*., Supplementary Figure S10
